# Targeting the protein–protein interaction between the CDC37 cochaperone and client kinases by an allosteric RAF dimer breaker

**DOI:** 10.1016/j.jbc.2025.111018

**Published:** 2025-12-08

**Authors:** Alison Yu, Shrhea Banerjee, Sravani Malasani, Bamidele Towolawi, Zhiwei Liu, Zhihong Wang

**Affiliations:** Department of Chemistry & Biochemistry, Rowan University, Glassboro, New Jersey, USA

**Keywords:** BRAF, CDC37, HSP90, allosteric kinase inhibitor, αC helix–β4 loop, client kinase, protein degradation

## Abstract

CDC37, a selectivity cochaperone in the heat shock protein (HSP90) chaperone machinery, plays a crucial role in facilitating recognition of client kinases and aiding their folding and maturation. RAF kinases, central components of the mitogen-activated protein kinase signaling pathway, rely on their interaction with CDC37 for stability and function. The RAF dimer interface, a key determinant of RAF kinase activity, overlaps with the CDC37-kinase client recognition motif known as the αC helix–β4 loop region. Here, we report that braftide, a peptide originally designed as a potent allosteric RAF kinase dimer disruptor, also triggers proteasome-mediated degradation of RAF kinases through a previously unclear mechanism. This study elucidates the mechanism underlying braftide’s dual functionality and evaluates the potential of targeting kinase-chaperone interactions in cancer cell lines. Using coimmunoprecipitation and NanoBiT assays, we confirmed braftide’s ability to selectively disrupt the CDC37-client kinase interaction while sparing HSP90. Through deuterium exchange mass spectrometry, molecular dynamics simulations, and in vitro crosslinking analyses, we mapped braftide’s binding region within the BRAF kinase domain and identified the CDC37 region implicated in client kinase association. Disruption of this interaction destabilizes RAF kinase clients, resulting in proteasomal degradation, reduced cellular proliferation, and increased apoptosis in cancer cell lines. Furthermore, braftide exhibits synergy with HSP90 inhibitors, jointly destabilizing CDC37-RAF complexes and HSP90. Our work identifies the αC helix–β4 loop as a novel allosteric site for targeting kinase–chaperone interactions and demonstrates the feasibility of disrupting the CDC37-client kinase interaction as an innovative therapeutic strategy.

Heat shock protein 90 (HSP90), an ATP-dependent protein chaperone, is involved in the folding, maturation, and activation of client proteins, with selectivity directed by cochaperones like CDC37 (1). CDC37 is well established as a kinase cochaperone that stabilizes kinases in a partially unfolded state (2, 3). CDC37 is responsible for recognizing HSP90 clients by assessing the thermal stability of the client kinase (4). Subsequently, the CDC37–kinase domain (KD) complex engages with the HSP90 molecular chaperone (5). The CDC37–HSP90 complex ensures proper kinase folding and stability, including that of key oncogenic proteins, such as BRAF, AKT, and HER2 (1, 4, 6). Disrupting this complex presents a novel therapeutic avenue. Inhibitors of HSP90 disrupt the HSP90 ATPase protein chaperone cycle, leading to proteasomal degradation of oncogenic proteins (6, 7, 8). However, direct targeting of HSP90 has proven challenging because of low selectivity and high cytotoxicity, with over 30 ATP-competitive inhibitors failing to gain Food and Drug Administration approval (9, 10). Notably, CDC37 is largely dispensable in most normal tissues, offering a potential therapeutic window for anti-CDC37 cancer therapies. CDC37 is frequently overexpressed in multiple cancer types to promote the maturation of mutated or overexpressed protein kinases (11, 12). Inhibiting CDC37 has been shown to selectively destabilize protein kinases CDK4/6 and enhance the efficacy of existing kinase inhibitors in tumor cells (13). Recent structural studies of HSP90–CDC37–client kinase complexes suggest an alternative approach to selectively degrade oncogenic kinases by targeting the CDC37–client interface, rather than directly inhibiting HSP90 (8, 10, 14, 15, 16, 17). Despite this promising approach, no inhibitors have yet been developed to efficiently disrupt the interaction.

The RAF kinase family, comprising ARAF, BRAF, and CRAF, is one of the well-studied clients of CDC37. RAF kinase is a core component of the mitogen-activated protein kinase (MAPK) (RAS–RAF–mitogen-activated protein kinase kinase [MEK]–extracellular signal–regulated kinase [ERK]) pathway, governing cell proliferation, survival, senescence, differentiation, and migration (18). BRAF, the most active RAF isoform, is dysregulated in 8% of all cancers and is intensely researched because of its role in oncogenesis (18). While ATP-competitive inhibitors like vemurafenib, dabrafenib, and encorafenib have shown success in treating BRAF V600E/K-mutant melanoma, they can lead to paradoxical activation of downstream ERK under certain conditions (19). This is linked to enhanced RAF dimerization, RAS–GTP association, and MEK binding (20, 21, 22), necessitating alternative therapeutic strategies.

To overcome these limitations, braftide (TAT-miniPEG-TRHVNILLFM) (23), a peptide developed to disrupt RAF dimerization by targeting the conserved dimer interface (DIF) among the RAF kinase family, along with other reported peptides (20, 24), demonstrates potent efficacy in downregulating the MAPK pathway. The sequence of braftide mirrors the residues spanning the tail end of the αC helix and a region known as the αC helix–β4 loop, which includes three key residues: BRAF R509, L515, and M517, critical for RAF dimerization. Braftide not only inhibits RAF kinase activity but also mitigates paradoxical activation induced by ATP-competitive inhibitors, both *in vitro* and in cells (23). Unexpectedly, braftide induces protein degradation of RAF kinases, a novel mechanism reminiscent of proteolysis targeting chimera–mediated selective protein degradation. Protein degradation offers a promising approach to achieve sustained and robust inhibition of protein function. Targeting mutant BRAF using proteolysis targeting chimeras has demonstrated degradation of mutant BRAF as a viable strategy to target the oncogenic kinase (25, 26, 27). The intriguing observation of protein degradation prompted us to investigate the underlying mechanism.

RAF family members are known clients of the HSP90–CDC37 chaperone complex and rely on their interaction with the CDC37–HSP90 chaperone complex to maintain basal protein stability (4). Prior biochemical studies, along with B/CRAF structure in complex with CDC37–HSP90, suggest that the αC helix–β4 loop (BRAF residues 510–520), a segment of the DIF, serves as the recognition site for CDC37 (15, 16, 28). Although the HSP90 and CDC37 interface is vast and dynamic (7), the αC helix–β4 loop is a well-defined structural motif conserved across RAF and other kinases (29). The RAF DIF, being involved in CDC37 association, dimerization, and activation, emerges as an attractive therapeutic target. Our previous work demonstrated that braftide, which inhibits the DIF, induces the degradation of BRAF–CRAF protein levels through the ubiquitin-26s proteasomal degradation system (23). We propose that braftide disrupts RAF–CDC37 interactions, thereby reducing RAF protein levels and offering a new strategy for targeting RAF-driven oncogenesis.

This study investigates braftide's ability to modulate the interaction between CDC37 and RAF kinases, exploring its role in promoting RAF degradation. We employ a combination of *in vitro* and cellular assays to demonstrate that braftide destabilizes RAF through the ubiquitin–proteasome system and impairs cancer cell survival. Our findings suggest that disrupting the CDC37–client kinase interaction represents a viable alternative to direct HSP90 inhibition. Braftide's ability to selectively disrupt the CDC37–RAF interaction, while sparing HSP90, provides an alternative and potentially more selective therapeutic strategy for targeting RAF and other oncogenic kinases.

## Results

### Braftide disrupts the interaction between the client RAF kinase and the CDC37 cochaperone

**Figure 1:**
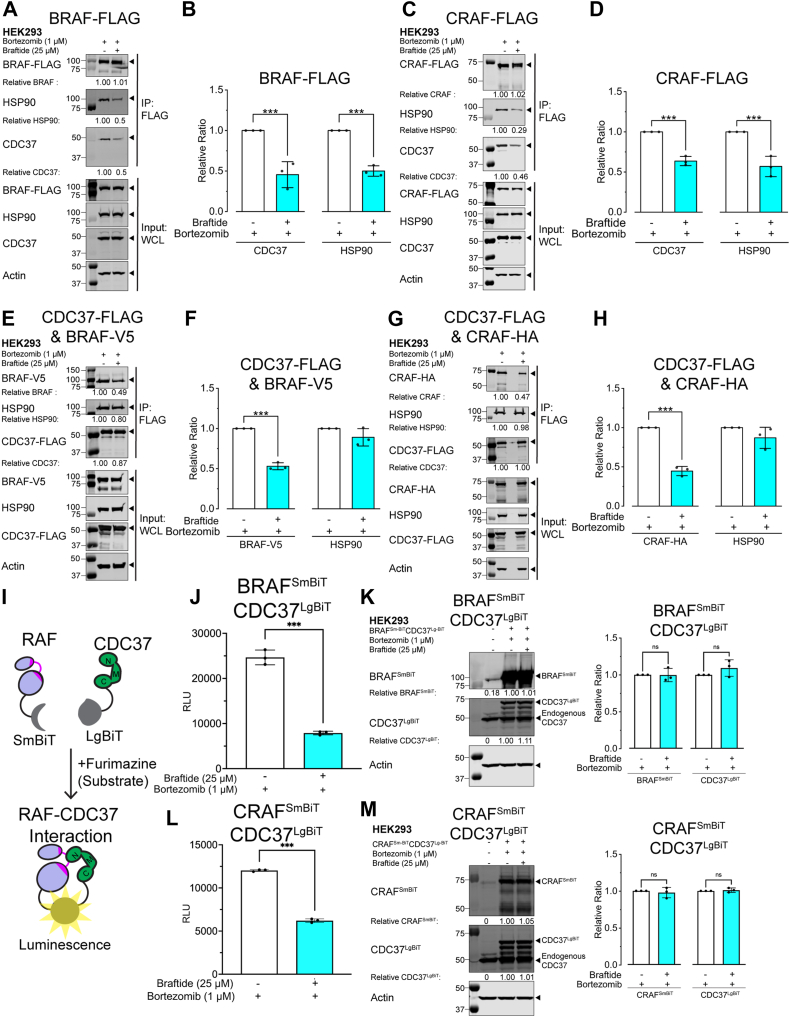
**Braftide disrupts the interaction between the client RAF kinase and the CDC37 cochaperone.***A*–*D,* BRAF-FLAG (*A* and *B*)/MBP-CRAF-FLAG (*C* and *D*) exogenously expressed in HEK293 cells in the absence and presence of 50 μM braftide treatment (4 h). Representative immunoblot of immunoprecipitated (IP) B/CRAF-FLAG for the coimmunoprecipitated (co-IP) CDC37 and HSP90 chaperone complex. *B* and *D,* densitometry analysis of CDC37 and HSP90 normalized to immunoprecipitated BRAF (*B*)/CRAF (*D*) across three biological replicates. *E*–*H,* CDC37-FLAG coexpressed with BRAF-V5 (*E* and *F*) or CRAF-HA (*G* and *H*). Representative blot of three biological replicates of immunoprecipitated CDC37-FLAG for co-IP BRAF-V5 (*E*) or CRAF-HA (*G*) and HSP90 in the absence and presence of braftide treatment (25 μM) in HEK293 cells. *I,* schematic illustrating NanoBiT luminescence assay. *J* and *L,* NanoBiT assay in the absence and presence of braftide (25 μM, 4 h) in HEK293 cells expressing NanoBiT constructs of BRAF^SmBiT^–CDC37^LgBiT^ (*J*) and CRAF^SmBiT^–CDC37^LgBiT^ (*L*). *K* and *M,* representative immunoblot of HEK239 cells expressing BRAF^SmBiT^–CDC37^LgBiT^ (*K*) and CRAF^SmBiT^–CDC37^LgBiT^ (*M*) in the absence and presence of braftide. *K*–*M,* densitometry analysis of CDC37^LgBiT^ and BRAF^SmBiT^ (*K*) or CRAF^SmBIT^ (*M*) normalized to no treatment control across three biological replicates. At least three independent biological replicates were performed for each experiment. HEK293, human embryonic kidney 293 cell line; HSP90, heat shock protein 90; LgBiT, large BiT; SmBiT, small BiT.

A coimmunoprecipitation (co-IP) strategy was employed to monitor the association of BRAF and CRAF with CDC37 and HSP90 in the absence and presence of braftide treatment. Our previous work revealed that braftide treatment triggers proteosome-mediated degradation of BRAF and CRAF; therefore, all the cells were pretreated with bortezomib, a proteosome inhibitor, prior to braftide treatment (Fig. S1, *F* and *G*), unless noted otherwise (23). BRAF-FLAG or MBP-CRAF-FLAG (herein called CRAF-FLAG) was transiently transfected in human embryonic kidney 293 (HEK293) cells and immunoprecipitated for RAF's association with the endogenous chaperone complex (Fig. 1, *A*–*D*). Notably, both braftide-treated BRAF and CRAF reduced their association with CDC37 and HSP90 compared with the nontreated control (Fig. 1, *A*–*D*). Braftide effectively disrupts BRAF–CRAF's association with the CDC37–HSP90 chaperone complex.

To further investigate whether this disruption specifically targets CDC37 or HSP90, we cotransfected CDC37–FLAG with BRAF-V5 or CRAF-HA in HEK293 cells and immunoprecipitated to assess CDC37–FLAG's interaction with RAF (BRAF-V5 or CRAF-HA) and HSP90. Braftide treatment reduced the CDC37–client BRAF–CRAF complex while maintaining the CDC37–HSP90 interaction (Fig. 1, *E*–*H*). Similar results were observed in the activating KRAS-mutant colon cancer cell line HCT116, where braftide reduced the CDC37–RAF interaction at lower doses than in HEK293 cells, suggesting this effect is not cell line specific (Fig. S1, *A*–*E*).

An orthogonal NanoBiT assay was developed to monitor the CDC37–RAF interaction in live cells (Fig. 1*I*). RAF and CDC37 are fused to two structurally complementary subunits of the NanoBiT enzyme, the small (Sm) and large (Lg) BiT. Structural complementation of the NanoBiT enzyme enables enzyme activity on furimazine, the substrate, and subsequently, a luminescence-based reporter of the CDC37 and RAF kinase interaction. Both NanoBiT components, CDC37-LgBiT and RAF-SmBiT, were coexpressed in HEK293 cells in the absence and presence of braftide treatment. Braftide treatment led to a reduction in luminescence, reflecting a decrease in the CDC37–RAF interaction (Fig. 1, *J* and *L*), whereas protein levels remained the same with bortezomib pretreatment (Fig. 1, *K* and *M*). Both the NanoBiT and IP CDC37–RAF interaction assays confirm that braftide disrupts the CDC37–RAF interaction in cells.

### Braftide directly binds to CDC37 and RAF, inducing proteasomal degradation of RAF *via* the ubiquitin–proteasome pathway

**Figure 2:**
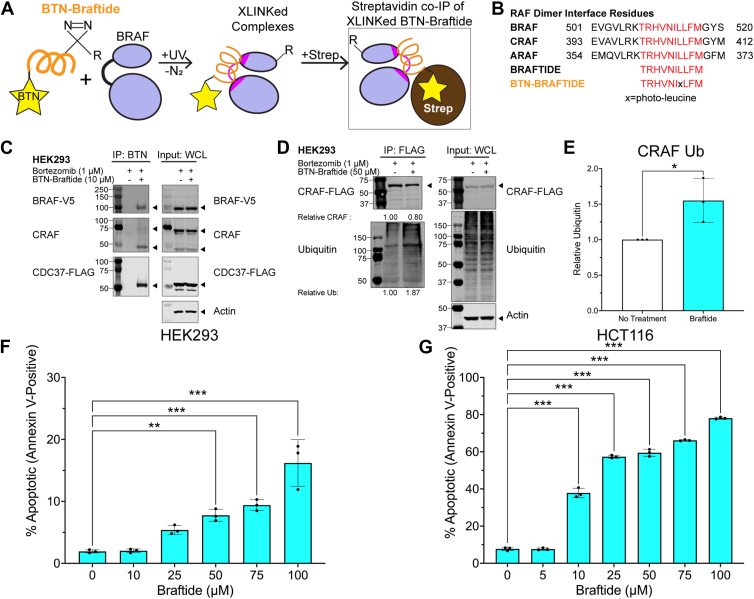
**Braftide binds to CDC37 and RAF kinases and induces proteasomal degradation of the client kinase *via* the ubiquitin–proteasome pathway.***A* and *B, cartoon schematic* of photo-activated crosslinking (XLINK) strategy of biotinylated (BTN)–braftide (*B*) with BRAF. Crosslinked BTN–braftide complexes are coimmunoprecipitated *via* streptavidin magnetic resin for further analysis. *C,* representative immunoblot of overexpressed BRAF-V5 and CDC37-FLAG in HEK293 cells in the absence and presence of 10 μM BTN–braftide and then immunoprecipitated and probed for individual proteins. *D* and *E,* representative immunoblot (*D*) and densitometry (*E*) of overexpressed MBP-CRAF-FLAG immunoprecipitated and probed for ubiquitination in HEK293 cells in the absence and presence of 50 μM braftide. At least three independent biological replicates were performed for each experiment. *F* and *G,* braftide-mediated CDC37 disruption triggers apoptosis in HEK293 (*F*) cells and HCT116 (*G*) cells treated with indicated concentrations of braftide for 4 h. Cells were stained with Annexin V, an apoptosis marker, and sorted *via* flow cytometry (n = 3). HEK293, human embryonic kidney 293 cell line.

To identify proteins interacting with braftide in cells, a crosslinking strategy was employed using biotinylated braftide with a photo-active leucine analog, herein called BTN-braftide, in HEK293 cells cotransfected with CDC37 and BRAF (Fig. 2*A*). This photo-activatable leucine within BTN-braftide contains a UV-responsive diazirine modification, enabling covalent bond formation with nearby amino acids upon UV exposure (30). Braftide contains three critical dimerization residues (BRAF amino acids R509, L515, M517) located in the RAF dimerization interface (23), conserved among all RAF family members (Fig. 2*B*). The modified leucine in BTN-braftide corresponds to residue 514 in the BRAF KD, a position not involved in critical interactions in available structural data or in prior alanine scanning of the DIF (24). Therefore, this alteration is not expected to affect the binding profile of braftide.

After UV light exposure, cells were collected, and the lysates were subjected to IP using streptavidin magnetic resin, followed by probing for BRAF, CRAF, CDC37, HSP90, and AKT1. BTN-braftide coimmunoprecipitated CDC37, BRAF, and endogenous CRAF (Fig. 2*C*). AKT1 is a well-established client kinase of CDC37, thus was selected as a control (4). Neither AKT1 nor HSP90 was pulled down in this assay. Our results also demonstrate that BTN–JAK2, a control peptide derived from the αC helix–β4 loop of the JAK2 kinase and sharing the same TAT-miniPEG fragment as braftide, was used to treat HEK293 cells cotransfected with CDC37 and RAF. This peptide did not induce protein degradation or reduce pERK levels (Fig. S2*A*). Accordingly, BTN–JAK2 served as a negative control and did not coimmunoprecipitate BRAF, CDC37, or HSP90 under the same conditions (Fig. S2*B*). Together, these findings indicate that braftide selectively interacts with CDC37 and RAF and that this interaction is not attributable to the TAT-miniPEG fragment.

Dissociating the client kinase from CDC37 disrupts its interaction with the HSP90 chaperone. CRAF is reliant on its association with HSP90 and CDC37 for stability and function (4). Given that braftide dissociates the CDC37–client interaction, we hypothesize that CRAF dissociation from CDC37 would increase CRAF instability, leading to its degradation *via* the ubiquitin–proteasome pathway. This hypothesis was tested by monitoring CRAF ubiquitination in HEK293 cells expressing MBP-CRAF-FLAG, pretreated with bortezomib to inhibit proteasomal degradation, both in the absence and presence of braftide treatment. Braftide treatment led to increased CRAF ubiquitination (Fig. 2, *D* and *E*). These findings support our hypothesis that the dissociation of client kinase from the CDC37 cochaperone destabilizes the client kinase, leading to subsequent degradation *via* the ubiquitin–proteasome pathway (23).

Previous studies have shown that knockdown of CDC37 expression *via* siRNA increases cellular apoptosis because of the degradation of client kinases in HCT116 and PC3 cells (31). Based on the observed increase in CRAF ubiquitination, we rationalized that braftide-mediated dissociation of RAF kinases from CDC37 would similarly promote cellular apoptosis. HCT116 cells and HEK293 cells were subjected to increasing concentrations of braftide (0, 5, 10, 25, 50, 75, and 100 μM) and stained for Annexin V, a marker of apoptosis. In both cell lines, braftide triggered dose-dependent apoptosis by disrupting the CDC37–RAF interaction (Figs. 2, *F* and *G*, S3). Notably, HCT116 cells showed higher sensitivity to braftide compared with HEK293, likely because of their higher endogenous expression level of BRAF and CDC37 (Fig. S4).

### Mapping the footprint of braftide on BRAF KD and CDC37

**Figure 3:**
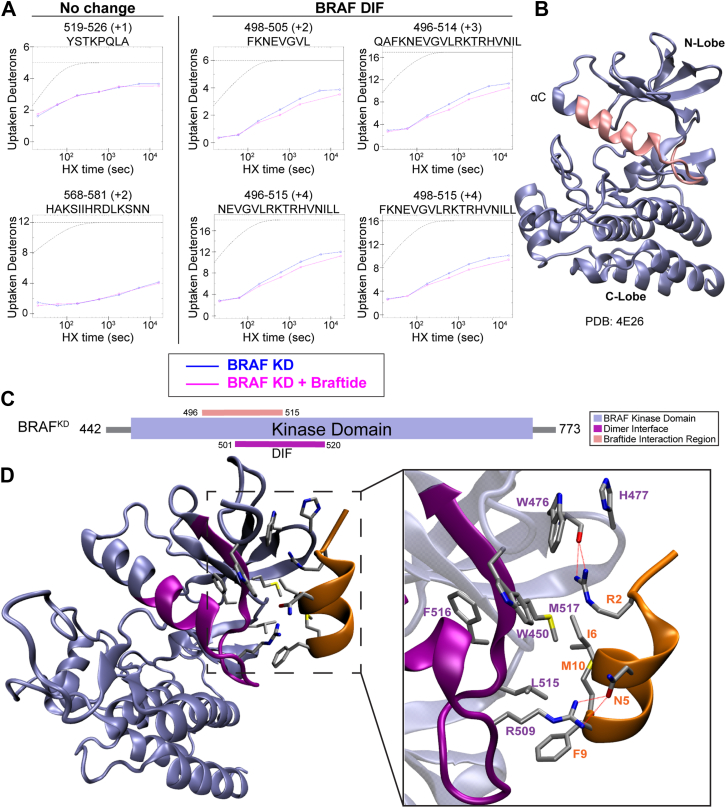
**Braftide interacts with the BRAF kinase domain *via* the αC helix–β4 loop of the dimer interface (DIF).***A,* representative BRAF^KD^ peptides identified by hydrogen–deuterium exchange mass spectrometry (HDX–MS) in the absence (*blue*) and presence (*pink*) of braftide. The peptide plots display slowed deuterium uptake, indicative of a broader trend across multiple overlapping peptides localized to the DI. *Gray dashed lines* represent theoretical exchange profiles: the *upper line* corresponds to a fully unstructured peptide, whereas the *lower line* represents a peptide with complete hydrogen bond protection. *B,* slowed deuterium uptake mapped onto the BRAF^KD^ crystal structure (Protein Data Bank [PDB] code: 4E26), highlighting regions affected by braftide (*pink*). *C,* braftide interaction region on BRAF^KD^ (*pink*), according to HDX–MS, overlaps with the DIF (*purple*). *D,* braftide (*orange*) binding to BRAF^KD^, MD simulation–predicted interacting residues are shown in *licorice representation.* BRAF^KD^ DIF region, identified in the crystal structure (PDB code: 4E26), highlighted in *purple*. MD, molecular dynamics.

The crosslinking experiment (Fig. 2*C*) captured the interaction between braftide and BRAF–CDC37 in HEK293 cells. We used hydrogen–deuterium exchange mass spectrometry (HDX–MS) to identify specific interaction regions between braftide and BRAF, providing insights into braftide's mechanism of disrupting RAF–CDC37 interaction. In these experiments, BRAF KD was incubated with and without braftide, followed by exposure to deuterated buffer at specific time points (20 s, 1 min, 3 min, 10 min, 30 min, 1.5 h, and 4.5 h) to allow hydrogen exchange in solvent-accessible regions. After the exchange reaction, the samples were quenched, digested into peptides, and analyzed using mass spectrometry (MS). Changes in deuterium incorporation between the braftide-bound and unbound BRAF samples were quantified, with regions showing reduced deuterium uptake indicating potential binding sites covered by braftide, as observed in duplicate HDX–MS experiments (Figs. 3*A*, S5). The entire BRAF KD sequence was covered in this experiment (Fig. S5), allowing comprehensive analysis of deuterium uptake differences, which were then mapped onto the BRAF structure (Fig. 3*B*) to reveal specific interaction regions associated with braftide binding. The identified binding region, spanning amino acids 496 to 515, overlaps with the BRAF DIF (Fig. 3*C*, *pink*), supporting the hypothesis that braftide targets this interface in its role as a dimer breaker.

In parallel, molecular dynamics (MD) simulations were carried out to model the interaction between the BRAF KD and braftide. The starting structure replicates interaction in the BRAF dimer (4E26) with braftide overlaying residues 508 to 517 of one monomer (Fig. S7, *A* and *B*). Analysis of the MD trajectory shows that the system undergoes significant conformational changes, quickly departing from the dimer-like interaction (Fig. S7, *C* and *D*). Nevertheless, the simulation eventually converged to a stable conformation with braftide forming a new set of contacts, including both hydrogen bonds and hydrophobic interactions, with residues at the BRAF DIF (Figs. 3*D*, S7*D*). The identified key interactions stabilizing the BRAF–braftide complex involve residues R509, L515, F516, and M517 from BRAF and R2, N5, I6, F9, and M10 from braftide (Figs. 3*D*, S7*A*). The MD results align with our HDX–MS data, which suggest that braftide binds at this critical region.

**Figure 4:**
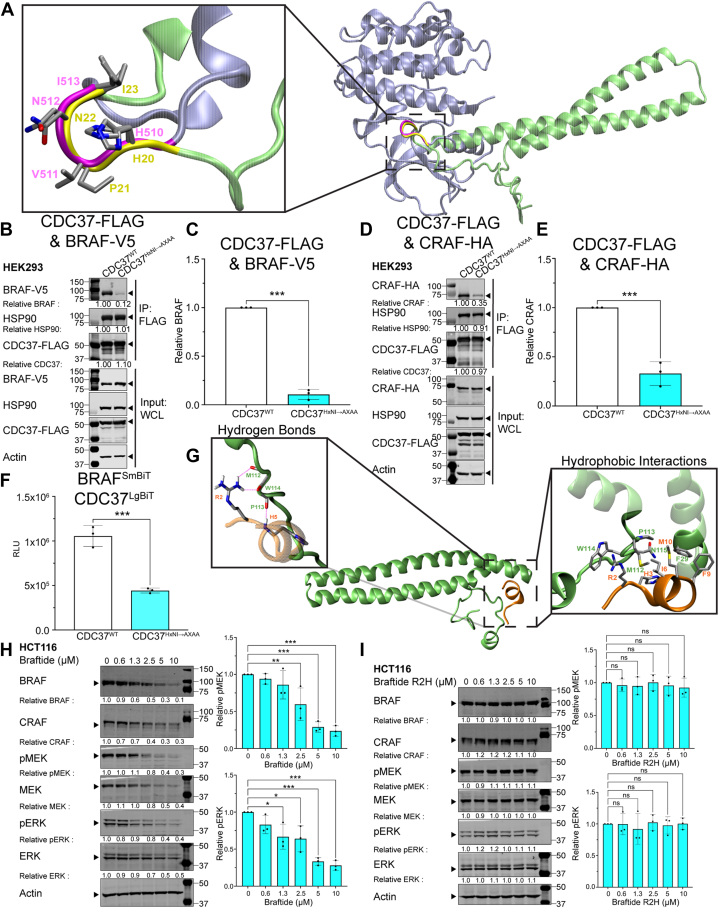
**The HxNI motif is a key interaction site between CDC37 and client kinases.***A,* overlay of the HxNI motifs of CDC37 (Protein Data Bank [PDB] code: 7ZR0) in *yellow* and BRAF^KD^ (PDB code: 4E26) in *magenta*. *B* and *E,* BRAF-V5 (*B* and *C*)/CRAF-HA (*D* and *E*) coexpressed with CDC37-FLAG^WT^ or CDC37-FLAG^HxNI→AxAA^ in HEK293 cells. Representative blot of three biological replicates of immunoprecipitated CDC37-FLAG for coimmunoprecipitated (co-IP) BRAF-V5 (*B*) or CRAF-HA (*D*) and HSP90. *C* and *E,* densitometry analysis of immunoprecipitated BRAF (*C*)/CRAF (*E*) across three biological replicates. *F,* NanoBiT assay with CDC37-FLAG^WT^ or CDC37-FLAG^HxNI→AxAA^ in HEK293 cells expressing NanoBiT constructs of BRAF^SmBiT^–CDC37^LgBiT^. *G,* interactions between CDC37 and braftide identified by MD simulation are shown in one overview (*middle*) and two zoomed-in views depicting molecular details of hydrogen bonds (*left*) and hydrophobic interactions (*right*). *H* and *I,* HCT116 cells treated with braftide (*H*) or braftide^R2H^ mutant (*I*) at the indicated concentrations for 4 h. Cells were harvested, lysed, and immunoblotted for MAPK proteins (BRAF, CRAF, MEK, phosphorylated MEK [pMEK], ERK, and pERK). Densitometry analysis of pMEK and pERK normalized to 0 μM braftide (*H*) or braftide^R2H^ (*I*). At least three independent biological replicates were performed for each experiment. ERK, extracellular signal–regulated kinase; HEK293, human embryonic kidney 293 cell line; HSP90, heat shock protein 90; MD, molecular dynamics.

Based on the structure of the CDK4 kinase in complex with the HSP90–CDC37, the αC helix–β4 loop has been proposed to serve as a potential molecular recognition motif for the CDC37 cochaperone (17). Vemurafenib binding to the ATP pocket of BRAF, known to stabilize the αC-helix OUT conformation of BRAF, disrupted the interaction between BRAF and CDC37 in the NanoBiT assay (Fig. S8, *A* and *B*), supporting the involvement of the αC helix–β4 loop of RAF kinase in mediating this interaction. By analyzing the structure of the BRAF–CDC37–HSP90 complex and overlaying the partially folded BRAF from this complex with a previously solved BRAF structure, we observed that the α helix–β loop of CDC37 superimposes on the αC helix–β4 loop of BRAF (Fig. 4*A*). The α helix–β loop of CDC37 contains the same HxNI motif (residue numbers H20, P21, N22, I23) as the αC helix–β4 loop of RAF kinase (residue numbers H510, V511, N512, I513), suggesting that CDC37 applies a similar mechanism to engage with the C-lobe of the protein kinase. This supports the hypothesis that the α helix–β loop in CDC37 is a potential site involved in mediating kinase–CDC37 interactions. To verify our hypothesis, we generated a CDC37 mutant in which residues H20, N22, and I23 were replaced with alanine. HEK293 cells were cotransfected with either BRAF-V5 or CRAF-HA and CDC37^HxNI→AxAA^-FLAG. Co-IP experiments pulling down BRAF or CRAF and probing for CDC37 revealed that the interaction between RAF and CDC37 was dramatically reduced upon mutation of the HxNI motif (Fig. 4, *B*–*E*). Consistent with these results, a NanoBiT assay showed that CDC37^HxNI→AxAA^ displayed markedly reduced association with BRAF, compared with wildtype (Fig. 4*F*).

MD simulations were also conducted to illustrate binding between braftide and CDC37 (Figs. 4*G*, S8, *C* and *D*). Two independent simulations with braftide initially positioned at very different sites on CDC37 converged to one single binding mode (Fig. S8, *C* and *D*). Braftide binds to a region on CDC37 (residue numbers F29, M112, P113, W114) in close proximity to the HxNI motif. To verify the interactions predicted by MD simulations, we synthesized a modified R/H peptide, in which the R2 residue of braftide, a key residue involved in interaction with CDC37, based on the simulations, was mutated to histidine. Unlike the unmodified braftide (Fig. 4*H*), the R/H peptide failed to induce protein degradation of BRAF, CRAF, MEK, and ERK and had no impact on MAPK signaling in HCT116 cells (Fig. 4*I*), verifying the key interactions between braftide and CDC37 observed in the MD simulations. This result reinforces the proposed mechanism by which braftide disrupts CDC37-mediated client stabilization.

### Braftide downregulates endogenous kinase levels *via* a structurally conserved αC helix–β4 loop interacting with CDC37

We propose that braftide targets CDC37 by binding close to the critical region for client kinase recognition, as shown in our MD simulations. Consequently, this interaction likely promotes the dissociation of CDC37 from a broader range of client kinases. This hypothesis is supported by the reduced association of CDC37 with AKT1, a well-characterized oncogenic kinase, upon braftide treatment (Fig. S9).

Our findings highlight the therapeutic potential of targeting CDC37–client kinase interactions, as oncogenic kinases are intrinsically unstable and thus are more dependent on their association with the HSP90–CDC37 chaperone complex for stability and activation compared with their wildtype counterparts (4). To assess the broader impact of braftide on kinase levels, we selected HCT116 cells, which exhibit high endogenous expression of CDC37 and are more sensitive to braftide treatment (Fig. S10). HCT116 cells harbor an upstream KRAS G13C–activating mutation, with a previously reported braftide EC_50_ of 7.1 ± 0.5 μM (23). To investigate differential protein expression, three independent biological replicates of HCT116 cells were treated with or without 10 μM braftide for 1 h, followed by protein profiling using LC–MS/MS. Proteomic analysis identified 5405 proteins (supporting information), of which 745 (13%) were differentially regulated with a cutoff of twofold change (Fig. S11). Among these, 627 proteins were downregulated (Fig. S11*A*), encompassing proteins with functions, such as catalytic activity, transferase, and hydrolase activity. Gene Ontology (GO) analysis revealed that biological processes significantly impacted by braftide included cellular and metabolic processes, signal transduction, and biosynthesis. While 118 proteins were upregulated, GO analysis indicated that catalytic activity and binding functions remained the most affected molecular functions among this subset (Fig. S11*B*).

**Figure 5:**
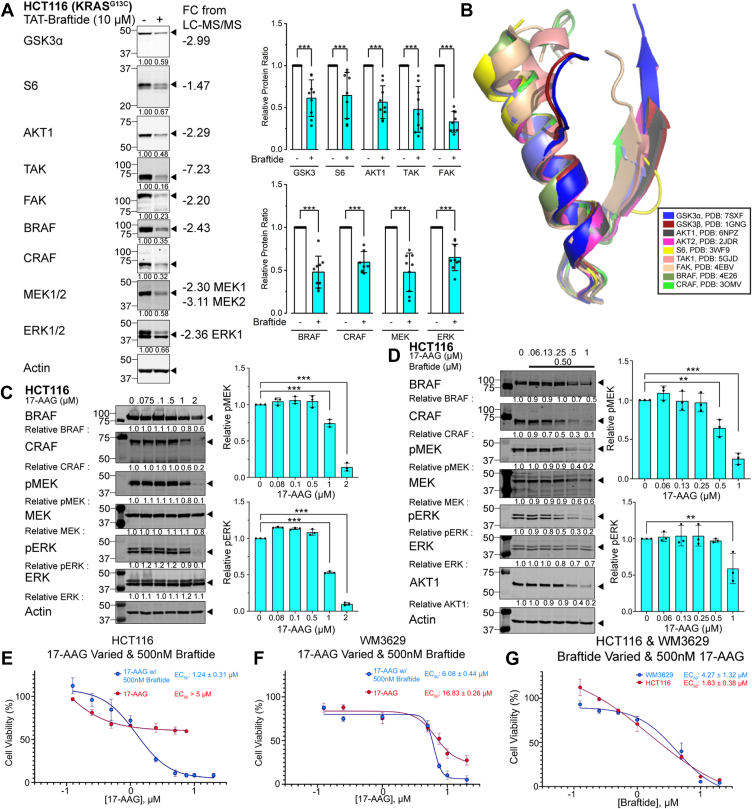
**Braftide downregulates endogenous kinase levels and synergizes with HSP90 inhibitor 17-AAG.***A,* representative immunoblot of selected kinases that are downregulated by braftide treatment. Densitometry analysis of nine biological replicates of downregulated proteins (n = 9). Graph bars represent the mean ± SD with corresponding *p* values (∗*p* < 0.05, ∗∗*p* < 0.01, and ∗∗∗*p* < 0.001). *B,* structural alignment of the conserved αC helix–β4 loop *via* PyMOL, with indicated Protein Data Bank (PDB) accessions. *C,* HCT116 cells were treated with 17-AAG at the indicated concentrations for 16 h. The cells were then immunoblotted for MAPK proteins. Densitometry analysis of pMEK and pERK normalized to no treatment (0 μM) 17-AAG (n = 3). *D,* HCT116 cells were treated with braftide and 17-AAG at the indicated concentrations. Concentration of braftide was held constant, and 17-AAG concentrations were varied. The cells were then immunoblotted for MAPK proteins (n = 3). Densitometry analysis of pMEK and pERK normalized to 0 μM 17-AAG and braftide. *E*–*G,* braftide and 17-AAG combination treatment inhibits cell proliferation in RAF dimer–dependent cancer cell lines, HCT116 (*E*) and WM3629 (*F*). *E,* HCT116 and (*F*) WM3629 cells were treated with 17-AAG at the indicated concentrations (0, 0.125, 0.250, 0.500, 1.0, 5, 7.5, 10, and 20 μM) and a constant braftide concentration (0.5 μM) for 24 h. Cells were treated with 17-AAG monotherapy at indicated concentrations for HCT116 (0, 0.125, 0.25, 0.5, 1, 2.5, 5, and 7.5 μM) and the same concentrations as combined therapy for WM3629. *G,* HCT116 and WM3629 cells were treated with braftide at the indicated concentrations (0, 0.125, 0.25, 0.5, 1, 5, 10, and 20 μM) and a constant 17-AAG concentration (0.5 μM) for 24 h. Cell viability was determined with the WST assay. EC_50_ values were obtained from a dose–response curve (four-parameter logistic equation). 17-AAG, 17-*N*-allylamino-17-demethoxygeldanamycin; ERK, extracellular signal–regulated kinase; HSP90, heat shock protein 90; MAPK, mitogen-activated protein kinase.

To validate the proteomic findings, several protein kinases identified as downregulated were selected for analysis *via* Western blotting, including GSK3α, S6, AKT1, TAK, and FAK, along with MAPK pathway members, BRAF, CRAF, MEK, and ERK. Consistent with the LC–MS/MS data (supporting information), treatment of HCT116 cells with 10 μM braftide for 1 h resulted in reduced protein expression levels of GSK3α, S6, AKT1, TAK, FAK, BRAF, CRAF, MEK1/2, and ERK1/2 (Fig. 5*A*). Among these, GSK3α, S6, FAK, BRAF, and CRAF have previously been identified as CDC37 client proteins (4). In parallel, we examined the effects of braftide treatment on several kinases identified as unchanged in protein levels by the proteomic analysis, including Chk2, PKCδ, and SRPK1. The results of these Western blot analyses (Fig. S12*A*) confirm that the protein levels of these kinases remain unchanged after braftide treatment, demonstrating that braftide exhibits selectivity for CDC37-regulated kinases.

The braftide-mediated downregulation of kinase expression aligns with the decreased kinase levels (AKT1, GSK3β, and CRAF) observed in siRNA-mediated CDC37 knockdown in HCT116 cells (31). The αC helix–β4 loop, a conserved element typically eight residues in length (with extensions in some kinases), anchors the N- and C-lobes through hydrophobic interactions and is one of the most stable structural elements in the KD (31). The structural conservation within the αC helix–β4 loop region among protein kinases (Fig. 5*B*) suggests that this motif may be a common interaction site between all client kinases and CDC37, serving as a recognition module for chaperone loading. Taken together, these findings suggest that braftide destabilizes the interactions between client kinases and the CDC37 cochaperone, leading to a reduction in protein kinase levels in HCT116 cells.

### Braftide synergizes with HSP90 inhibition to decrease cell viability

Braftide exhibits broad-spectrum effects on kinases that rely on CDC37 for stability and activation, providing a compelling basis for further exploration of CDC37 as a therapeutic target. Current efforts in HSP90 inhibition predominantly target ATP competitive inhibition to disrupt the chaperone cycle. However, this approach often requires high dosages, making cells more susceptible to thermal damage and inducing HSR1 expression (10). HSP90 inhibitors are limited by cytotoxicity arising from the inhibition of a numerous and diverse client base. ATP competitive inhibitors for RAF kinase encounter key drawbacks such as paradoxical activation and drug resistance (17, 32, 33). Given CDC37's role as a selectivity module for the HSP90 chaperone, we investigated whether disrupting the ternary complex *via* two approaches would synergize and promote cancer cell apoptosis. To overcome the limitations associated with RAF and HSP90 inhibitors, we propose inhibiting the ternary complex using braftide in combination with submaximal concentrations of 17-AAG (17-*N*-allylamino-17-demethoxygeldanamycin), an HSP90 inhibitor. While 17-AAG is a potent small-molecule HSP90 inhibitor, its clinical trials faced challenges because of high cytotoxicity (10). Leveraging braftide's capability to dissociate client kinase from the CDC37 cochaperone, we speculate that 17-AAG could synergize with braftide to reduce oncogenic kinase activity.

HCT116 cells were treated with 17-AAG alone or in combination with braftide (Fig. 5, *C* and *D*). HSP90 monotherapy (17-AAG) depleted CRAF kinase level at a concentration of 2 μM (Fig. 5*C*), whereas braftide monotherapy (23) depleted RAF kinase levels at a concentration of 10 μM. To investigate potential synergy, we assessed combinatorial treatments in HCT116 cells, using a fixed concentration of 500 nM of the primary drug (braftide) with increasing concentrations (0 to 1 μM) of the secondary drug (17-AAG) (Fig. 5*D*). Neither 17-AAG nor braftide monotherapy exhibited obvious inhibition at the concentration of 500 nM. Intriguingly, the combination of 500 nM of 17-AAG and 500 nM braftide resulted in significant degradation of CRAF kinase and downregulation of MAPK signaling, an effect unattainable with much higher concentrations of 17-AAG or braftide alone. This clear synergy was evident at submaximal concentrations of both reagents, necessary to attenuate the MAPK pathway through disrupting the ternary complex (Fig. 5*D*).

Based on the observed synergistic effects of inhibiting the ternary complex, the antiproliferative effects were explored using combinatorial treatment in two RAF-dimer–dependent cell lines: HCT116 and WM3629. HCT116 cells mainly rely on the wildtype BRAF–CRAF heterodimer and BRAF–BRAF homodimer to relay the MAPK signal, whereas WM3629 cells contain the BRAF D594G mutant, which is a kinase-dead mutant and depends on dimerization with CRAF for activation (34). The reported EC_50_ of braftide for WM3629 is 11.06 ± 1.12 μM (34). In HCT116 cells, the EC_50_ of 17-AAG in combinatorial treatment, with a constant braftide concentration of 500 nM and varied 17-AAG concentrations (0, 0.125, 0.250, 0.500, 1.0, 5, 7.5, 10, and 20 μM), was determined to be 1.2 ± 0.31 μM (Fig. 5*E*). In contrast, the EC_50_ value of 17-AAG in the absence of braftide exceeded 5 μM (Fig. 5*E*). Disrupting the CDC37–RAF complex in conjunction with 500 nM of braftide resulted in an EC_50_ of 6.06 ± 0.44 μM for 17-AAG in mutant BRAF WM3629 cells, whereas the EC_50_ value of 17-AAG in the absence of braftide approached 16.83 ± 0.26 μM (Fig. 5*F*). In both cell lines, we observed synergy between braftide and 17-AAG. In addition, in parallel, combination treatment with a constant 17-AAG concentration and varied braftide concentration yielded EC_50_ values of 1.63 ± 0.38 μM and 4.27 ± 1.32 μM for HCT116 and WM3629 cells, respectively (Fig. 5*G*). Annexin V staining was conducted to directly assess apoptosis in HCT116 cells treated with 17-AAG monotherapy and the combination of braftide and 17-AAG. The results demonstrate a significant increase in apoptotic cell death with the combination therapy compared with monotherapy (Fig. S12, *B* and *C*). This effect is particularly evident at lower doses of the combination therapy, underscoring its enhanced apoptotic potential. Braftide exploits the reliance of oncogenic kinase dependence on CDC37 and synergizes with 17-AAG to sensitize cells to submaximal dosage treatment.

## Discussion

**Figure 6:**
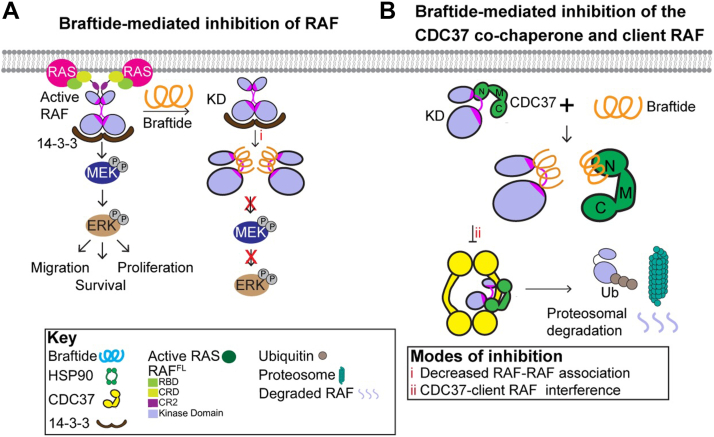
**Braftide disrupts RAF dimers and the CDC37–RAF client kinase complex.***A,* braftide binds to the dimer interface of RAF to dissociate RAF dimers. *B,* braftide binds to CDC37 to disrupt the cochaperone CDC37 with client kinases. Braftide treatment increases the ubiquitination of RAF, which leads to proteasomal-mediated degradation.

Our analyses have identified braftide's role in disrupting critical interactions within the cellular kinase regulatory network. Initially designed as an allosteric disruptor of RAF kinase dimerization (Fig. 6*A*), our analyses uncover the multifaceted nature of braftide, shedding light on its intricate mechanisms and therapeutic potential. Through HDX–MS, MD simulations, proteomics analyses, structural elucidation, crosslinking, live cell NanoBiT assays, and co-IP, we uncover braftide's secondary function (Fig. 6*B*). Braftide disrupts critical interactions for the stability and function of client kinases within the CDC37–HSP90 chaperone complex. By disrupting chaperone cycle entry, the client RAF kinase is destabilized, ubiquitinated, and degraded *via* the ubiquitin–proteasome pathway. Our data collectively reveal the mechanisms underlying braftide-triggered apoptosis in multiple cell lines, highlighting the therapeutic potential of targeting client kinase–CDC37 interactions in cancer cells.

The αC helix–β4 loop is a conserved structural feature among eukaryotic protein kinases and has been suggested to coordinate the cooperativity between the N-lobe and C-lobe. In RAF kinases, the αC helix–β4 loop is a critical component of the DIF, essential for activation. In addition, the αC helix–β4 loop has been deemed the recognition site of HSP90–CDC37, which is responsible for the proper folding of at least 60% of the human kinome. Our site-mutagenesis results suggest that the HxNI motif of CDC37 mimics the conformation of the αC helix–β4 loop and uses the same mechanism to form hydrophobic interactions with the C-lobe of protein kinases, thereby leading to unfolding of the N-lobe and its subsequent loading into the lumen of HSP90 for folding. Hotspot mutations have been identified on the αC helix–β4 loop, highlighting the significance and versatility of this structural motif. Despite its central role in kinase function and regulation, the therapeutic potential of the αC helix–β4 loop has remained largely unexplored. Braftide is the first reported chemical tool that disrupts interactions between client kinases and the CDC37 cochaperone. By mimicking the αC helix–β4 loop of RAF kinases, braftide not only disrupts RAF dimerization but also impairs the ability of CDC37 to interact with kinases. Our findings identify the αC helix–β4 loop as a key recognition site of CDC37, emphasizing its regulatory importance in kinase function folding and activation.

Our studies with braftide demonstrate that therapeutic agents targeting the αC helix–β4 loop could selectively inhibit aberrant kinase signaling implicated in various diseases, including cancer and inflammatory disorders. Targeting this loop offers the potential for selective protein degradation of oncogenic kinases, circumventing drug resistance mechanisms associated with traditional kinase inhibitors. By leveraging the principles of targeted protein degradation, braftide could serve as a prototype for designing molecules that promote the proteasomal clearance of dysregulated kinases driving oncogenesis. This also provides a great approach to target “undruggable” protein kinases, such as pseudokinases, which rely on these interactions for function. As a pioneering tool, braftide lays the foundation for further exploration of the αC helix–β4 loop as a therapeutic target. Future research into the structure, function, and evolutionary adaptations of this motif will provide deeper insights into kinase regulation and the discovery of novel, highly specific protein kinase inhibitors.

Unlike conventional HSP90 inhibitors, braftide preserves HSP90 activity while disrupting CDC37–client interactions, minimizing cytotoxicity and enhancing therapeutic precision. Braftide's ability to destabilize RAF through CDC37 inhibition may offer an alternative to high-dose HSP90 inhibitors, mitigating their associated side effects. Conventional HSP90 monotherapies, such as 17-AAG, prove ineffective because of their impact on a large HSP90 client base, leading to global cellular dysregulation (8). While most HSP90–CDC37 inhibitors focus on HSP90 ATP-competitive inhibition (6), emerging strategies suggest targeting CDC37 to circumvent the deleterious consequences of HSP90 inhibitors and indirectly inhibit the oncogenic kinase (8, 10). Current protein–protein interaction disruptors of the HSP90–CDC37 complex exclusively target HSP90 to allosterically dissociate the HSP90–CDC37 complex, as observed by conglobatin A and platycodin D (35). While disrupting the HSP90 and CDC37 interface interferes with substrate client protein maturation, this mode of modulation also affects HSP90 activity (6, 36). Braftide emerges as the first chemical probe disrupting the CDC37–client kinase interface while preserving HSP90 activity, with the potential to synergize with HSP90 inhibition and reduce cell proliferation. Our studies illustrate the promising effect of combining braftide along with existing HSP90 inhibitors as a viable avenue to combat oncogenic kinase signaling by targeting the ternary complex and disrupting crucial kinase regulatory mechanisms. This dual inhibition minimizes promiscuity within large HSP90 client bases. These analyses form the foundation for refining peptides into small molecular inhibitor(s) targeting the CDC37 cochaperone and dysregulated kinases for monotherapy as well as combined use with HSP90 inhibitors.

Braftide serves as both a powerful research probe and a prototype therapeutic agent, paving the way for targeted protein degradation strategies to combat kinase-driven diseases. Braftide represents a promising allosteric proof-of-concept disruptor of the CDC37–client kinase interaction and demonstrates the feasibility of targeting the association of client kinase with the chaperone complex. The development of peptide-guided small molecular inhibitors has demonstrated success, exemplified by a bespoke small molecule derived from the template macrocyclic peptide 1 to target nicotinamide *N*-methyltransferase for enzymatic inhibition in cells (37). Similar approaches should be employed to design CDC37–client small molecular disruptors, focusing on braftide mimetics to enhance selectivity, stability, and cell permeability in these inhibitors. Future efforts aimed at transforming braftide into a small molecule should prioritize enhancing selectivity for both the CDC37 cochaperone and the oncogenic kinase. MS revealed a broad spectrum of kinases impacted by braftide, suggesting braftide interacts and inhibits CDC37 function beyond RAF kinase. Future studies should develop inhibitors that target specific pairs of CDC37–client kinases to achieve higher selectivity and reduced toxicity. Achieving a structure of braftide in complex with BRAF and CDC37 will be instrumental in pursuing these objectives and guiding the development of more precise therapeutic intervention.

While this study emphasizes the impact of braftide-targeted chaperone deprivation on RAF kinases, we acknowledge that similar effects are anticipated for other client protein kinases. This has been evidenced through our global proteomic analyses and cell-based assays. However, this does not diminish the significance of using braftide as a chemical probe to explore the potential antagonism of oncogenic kinases beyond RAF kinases, aiming to achieve strong antitumor activities by depleting these oncogenic client protein kinases, such as CDK4, AKT, among others. Targeting CDC37 is believed to be superior to HSP90-directed inhibition, as it spares nonkinase HSP90 clients, such as the steroid hormone receptors. While HSP90 plays diverse roles in various protein categories, CDC37 primarily functions in the biological functionality, stability, and regulation of protein kinases. The specificity underlying this preference is highly interesting, yet remains largely unexplored because of the lack of suitable chemical probes.

## Experimental procedures

### Peptides

TAT–Braftide (GRKKRRQRRRPQ-miniPEG-TRHVNILLFM), BTN–Braftide (Biotin-GRKKRRQRRRPQ-miniPEG-TRHVNILxFM), TAT–Braftide^R2H^ (GRKKRRQRRRPQ-miniPEG-THHVNILLFM), TAT–JAK2 (negative control peptide) (GRKKRRQRRRPQ-miniPEG-SLQHDNIVKY), and BTN–JAK2 (Biotin-GRKKRRQRRRPQ-miniPEG-SxQHDNIVKY), where x is L-Photo-Leucine (ThermoFisher; 22610) was purchased from Lifetein with TFA removal. The purity was determined through HPLC (>95%) and confirmed through MS.

### Plasmids

6X-HIS-BRAF-WT/FLAG or V5 was prepared as previously described (34, 38), and MBP-CRAF-FLAG was created using common cloning procedures with pcDNA 4/TO (Invitrogen) as the vector. CDC37-myc-FLAG was purchased from Origene (RC201002).

### Protein purification

The 6XHIS-BRAF KD (residues 442–723, supplementary information 1) was purified as previously reported (39) with the human sequence expressed in *Escherichia coli*. The KD contains 16 solubilizing mutations (I543A, I544S, I551K, Q562R, L588N, K630S, F667E, Y673S, A688R, L706S, Q709R, S713E, L716E, S720E, P722S, and K723G), which were determined to preserve kinase activity and increase protein expression in *E. coli*. Mutations of the 16 residues are not involved in MEK1 interactions (40, 41). BRAF KD was expressed in *E. coli* BL21 codon+ cells at 37 °C until an absorbance between 0.6 and 0.8. Protein induction was induced by 1 mM IPTG, 16 °C in a shaking incubator, 200 RPM. The pellet was lysed in a buffer containing 50 mM Hepes (pH 7.4), 250 mM NaCl, 50 mM KCl, 10 mM imidazole, 10 mM β-mercaptoethanol, 10% glycerol, and 1X protease inhibitor (Roche) under sonication. The lysate was clarified *via* centrifugation and subjected to purification through nickel resin and eluted by increasing imidazole (50–250 mM) concentrations. The protein was concentrated (Millipore) and further purified *via* size-exclusion chromatography (Superdex 200). Protein fractions were analyzed on SDS-PAGE, pooled, and flash frozen for analytical use.

### Global proteomic identification of braftide-regulated proteins

HCT116 cells were treated in the absence and presence of 10 μM TAT–braftide, 1 h, 37 °C. Cell pellets were harvested in PBS and cryopreserved. Samples were sent to be lysed and identified through MS in three biological replicates (LC–MS/MS; The Wistar Institute). Data were processed through the previously reported GO analysis *via* the Panther database (42) and plotted on Origin (OriginLab Corporation). For the volcano plot, the cutoff for the presented data is *p* = 0.01 (∗∗), *q* value <0.05 (Student's *t* test value adjusted for multiple comparisons using the Benjamini–Hochberg false discovery rate correction) and detected in at least two or more replicates. A total of 5405 proteins were identified, emphasizing a twofold change in NeonGreen and in red. Source data are available in the supporting information files.

### Transient transfection into mammalian cells

HEK293 cells were seeded at 1(10)^6^ cells per well for a 6-well plate or 5(10)^6^ for 10 cm dishes and incubated until 40% to 60% confluency. Transfection was carried out using a 1:3 DNA:PEI-MAX ratio in OPTI-MEM and allowed 48 h for protein expression. For peptide assays, 48 h post-transfection, cells were treated with TAT–braftide carried in OPTI-MEM at indicated concentrations. Cells were harvested in cold PBS. Harvested cells were lysed in 4% SDS or modified radioimmunoprecipitation assay (RIPA) for co-IP experiments. Quantification of total protein by the bicinchoninic acid assay was carried out according to the manufacturer's protocols (Pierce; catalog no.: 23225).

### Co-IP in cells

Transfected HEK293 cells were treated with the indicated treatments and harvested in cold PBS. The cell pellets were lysed in modified RIPA buffer (50 mM Hepes [pH 7.4], 150 mM sodium chloride, 0.1% NP-40 [IGEPAL630], 1 mM EDTA, 5% glycerol, 1 mM PMSF, 20 mM β-glycerophosphate, 2.5 mM sodium pyrophosphate, and 1 protease inhibitor tablet) and incubated with rotation for 2 h, 4 °C. Cell lysates were then incubated with resin (FLAG-M2 magnetic, Sigma [M8823] or streptavidin, Pierce [88816]) for 2 h to bind the tagged BRAF–CRAF or BTN–braftide-crosslinked proteins. The samples were washed five times in a modified RIPA buffer and quenched in loading dye (BTN IP elution was supplemented with 20 mM biotin in loading dye) and heated at 95 °C, 5 min. Samples were analyzed *via* immunoblot. Antibodies used in this study are listed in Table 1. Treatment of cells was indicated in the figure legends.

**Table 1:** Antibodies used in immunoblots

Manufacturer	Catalog number	Name
Sigma	F1804	FLAG
Cell Signaling	3724	HA
Cell Signaling	4694	MEK1/2
Cell Signaling	9154	pMEK
Cell Signaling	4696	ERK1/2
Cell Signaling	4370	pERK
Cell Signaling	2317	S6
Cell Signaling	3700	β-Actin
Cell Signaling	4877	HSP90
Cell Signaling	4222	CDC37
Cell Signaling	3285	FAK
Cell Signaling	9422	CRAF
Cell Signaling	14814	BRAF
Cell Signaling	2938	AKT1
Cell Signaling	4337	GSK3α
Cell Signaling	2289	PP5
Cell Signaling	5206	TAK1
Cell Signaling	3936	Ubiquitin
Cell Signaling	52012	SRPK1
Cell Signaling	2662	Chk2
Cell Signaling	2058	PKCδ
Genetex	42525	V5
Licor	926-32211	Anti-rabbit
Licor	926-68070	Anti-mouse

### Cloning of NanoBIT constructs

NanoBiT cytomegalovirus MCS BiBiT vector, which contains a BRAF N terminal fused with LgBiT and CRAF N terminal fused with SmBiT, was purchased from Promega. CDC37 LgBiT and CRAF SmBiT was generated using the standard Gibson Assembly (NEB HiFi Assembly) using NanoBiT BiBiT as the vector and following primers: 5′ CCACCTCCTCCGAGAGAAACCACACTGACATCCTTCTCA 3′, 5′ TTTTGCAGCTAGCGATCGCCATGGTGGACTACAGCGTG 3′, 5′ GGCGATCGCTAGCTGCAAAAAG 3′, 5′ GTTTCTCTCGGAGGAGGTG 3′, and CDC37 LgBiT and BRAF SmBiT was generated using the standard Gibson Assembly using NanoBiT BiBiT as the vector and following primers: 5′ CTATAGGGCTAGCGATCGCCATGGCGGCGCTGAGCGGTG 3′, 5′ CCACCTCCTCCGAGAGAAACGTGGACAGGAAACGCACC 3**′**, 5′ GTTTCTCTCGGAGGAGGTGG 3′, 5′ GGCGATCGCTAGCCCTATAG 3′. Primers were designed to introduce H20A, N22A, I23A triple mutation within the CDC37 FLAG tag and for NanoBiT construct CDC37(HNI/A) LgBit and BRAF SmBiT. Primers were designed to introduce the desired substitutions. The forward primer sequence was 5′ gccgccGACACGGCCAGTCTCTTC 3′, and the reverse primer sequence was 5′ gggggcCGTCTCGTCTTCATCATCAG 3′. Site-directed mutagenesis was carried out using the Q5 Site-Directed Mutagenesis Kit (NEB; E0554) following the manufacturer's protocol.

### NanoBiT luminescence CDC37–RAF interaction reporter assay

HEK293 cells were transiently transfected with BRAF^SmBiT^–CDC37^LgBiT^ or CRAF^SmBiT^–CDC37^LgBiT^ at 2 μg plasmid per well and incubated at 37 °C. Twenty-four hours post-transfection, cells were trypsinized, pelleted, resuspended in OptiMEM + 4% fetal bovine serum, and reseeded onto a 96-well plate at 5(10)^4^ cells per well and then incubated at 37 °C overnight. Cells were pretreated with 1 μM bortezomib for 5 h and then braftide for 4 h at 0 and 25 μM. After treatment, 10 μM furimazine substrate was added to each well, and luminescence readings were taken at 470 to 480 nm using a CLARIOstar plate reader. Statistical significance was determined through a paired *t* test where indicated, followed by Tukey's honest significant difference post hoc test with corresponding *p* values (∗*p* < 0.05, ∗∗*p* < 0.01, and ∗∗∗*p* < 0.001). All graph bars represent the mean ± SD with individual data points per biological replicate with corresponding *p* values.

### Crosslinking BTN–braftide and BTN–JAK2 to HEK293 overexpressed CDC37 and BRAF

HEK293 cells were cotransfected with CDC37 (FLAG-tagged) and BRAF (V5-tagged). Cells were harvested in cold PBS, and pellets were lysed in modified RIPA buffer with rotation for 2 h, 4 °C. Cell lysate (1 mg) was incubated with 0 or 10 μM BTN–braftide/JAK2, with rotation for 4 h, at 4 °C. Samples were then exposed to 365 nM UV light for 1 h over ice. After UV treatment, samples were incubated with Pierce Streptavidin magnetic beads (catalog no.: 88816) for 17 h at 4 °C, to bind BTN–braftide. The samples were washed five times in high salt wash buffer (20 mM Hepes [pH 7.4], 500 mM NaCl, and 5% glycerol) and quenched with 25 mM biotin in loading dye and 95 °C, 5 min. Samples were analyzed *via* immunoblot.

### Apoptosis assays

HEK293 cells and HCT116 cells were treated with either control or braftide at the indicated concentrations for 4 h. After treatment, both the floating and adherent cell populations were collected and adjusted with PBS (final concentration: 1 × 10^5^ cells/ml). The cells were then mixed with the Annexin V (Cytex; FCCH100108) reagent in a 5:1 ratio (100 μl of cells mixed with 20 μl of Annexin V reagent) and allowed to stain in the dark, at room temperature for 15 min. The cells were then analyzed on a Guava Muse Cell Analyzer (Millipore).

### Hydrogen–deuterium exchange MS

BRAF^KD^ (10 μM) in a buffer solution (20 mM Hepes [pH 7.4], 150 mM NaCl, and 5% glycerol in H2O), in the presence and absence of braftide (30 μM), was mixed with deuterated buffer (20 mM Hepes [pH 7.4], 150 mM NaCl, and 5% glycerol in D2O) at a 1:5 volume ratio (v:v) to initiate HDX. The exchange times varied from 20 s to 4.5 h. The reaction was quenched by mixing the sample 1:1 (v:v) with cold quench buffer (100 mM phosphate, pH 2.4, 0.5 M Tris(2-carboxyethyl)phosphine, 3 M guanidium chloride), lowering the pH to 2.4. The quenched solution was digested using an immobilized pepsin column, and the resulting peptides were trapped and desalted on a C8 column (Higgins Analytical TARGA C8, 5 μm, 5 × 1.0 mm). Peptides were desalted for 3 min at 0 °C before being eluted at 8 μl/min using a gradient of 10% to 40% acetonitrile over 15 min. The eluted peptides were passed through an analytical column (Higgins Analytical TARGA C8, 5 μm, 50 × 0.3 mm) and introduced into a THERMO Q-Exactive mass spectrometer *via* electrospray ionization. Peptides were identified through MS/MS analysis of nondeuterated samples, with data analyzed using SEQUEST (Thermo Proteome Discoverer) against a database containing sequences of BRAF^KD^, braftide, pepsin, potential contaminants, and decoy proteins. Deuterated samples were analyzed using ExMS2 software (developed by Englander Lab at University of Pennsylvania).

### Cell Viability

HCT116 and WM3629 cells were seeded onto polylysine-coated, clear-bottomed, 96-well plates at 15(10)^3^ cells per well and allowed to incubate for 24 h. Cells were treated with braftide and 17-AAG mixtures at indicated concentrations for 24 h (37 °C). The WST-1 cell viability assay was carried out according to the manufacturer's protocols (Roche; 11644807001) for 4 h, and absorbance readings were taken at 450 nM in a CLARIOstar plate reader. For the dose–response curves of braftide and 17-AAG, the data were fit to the 4-parameter logistic equation (dose–response fitting equation) in GraphPad Prism (Dotmatics):

$$y=A1+\left(A2-A1\right)÷\left(1+10^{\left(LOGx0-x\right)p}\right)$$

Error bars are indicative of the SEM of each point.

### MD Simulations

MD simulations were performed using the Amber22 software (43) package, with the FF19SB force field for protein (44). The initial structure of the BRAF KD–Braftide system was built based on the crystal structure of the active BRAF KD dimer (Protein Data Bank code: 4E26). Specifically, one KD monomer and the T508 to M517 (TRHVNILLFM) region of the other monomer were extracted (Fig. S7, *A* and *B*). For the CDC37–Braftide systems, the crystal structure of the CDC37–HSP90–BRAFV600E complex (Protein Data Bank code: 7ZRO) was used with braftide positioned in two different sites (Fig. S8*C*). Each system was solvated in a periodic box with explicit OPC water molecules and just the necessary number of counterions (Na^+^ or Cl^−^) to neutralize the overall charge of the system (45). Equilibration of the system followed a consistent protocol, which began with short minimization runs to fix possible bad contacts in the initial structure, proceeded to gradually heating the system to 300 K, then a constant pressure (NPT) simulation at 1 atm and 300 K to equilibrate the simulation box size to be consistent with the experimental density of an aqueous solution. Finally, a production run was performed in the NVT ensemble at 300 K for 1 μs, with a timestep of 1 fs. The trajectories were then analyzed, focusing on the structural features and conformational changes at the BRAF KD (or CDC37)–Braftide interface, with the cpptraj program in the Amber22 package.

### Immunoblot analysis

Western blot band densities were quantified with ImageJ software (National Institutes of Health) and normalized to the indicated loading control. Statistical significance was determined through one-/two-way ANOVA, where indicated, followed by Tukey's honest significant difference post hoc test with corresponding *p* values (∗*p* < 0.05, ∗∗*p* < 0.002, ∗∗∗*p* < 0.0002, and ∗∗∗∗*p* < 0.0001). All graph bars represent the mean ± SD with individual data points per biological replicate with corresponding *p* values. Antibodies used in this study are listed in Table 1.

## Data availability

All data supporting the findings of this study are available within the article and its supporting information files.

## Data sharing plan

All data are included in the article and supporting information.

## Supporting information

This article contains supporting information.

## Conflict of interest

The authors declare that they have no conflicts of interest with the contents of this article.
