# Supplementary material for: Targeting the protein–protein interaction between the CDC37 cochaperone and client kinases by an allosteric RAF dimer breaker
Source: J Biol Chem. 2025 Dec 8;302(2):111018. doi: 10.1016/j.jbc.2025.111018 (PMC12830207; doi:10.1016/j.jbc.2025.111018)
Supplement: Supplemental Figures and Information [file mmc1.docx]

**Supplemental Figures and Information**

**Targeting the Protein-Protein Interaction Between the CDC37 Co-Chaperone and Client Kinases by an Allosteric RAF Dimer Breaker**

Alison Yu^⊥^, Shrhea Banerjee^⊥^, Sravani Malasani, Bamidele Towolawi, Zhiwei Liu, and Zhihong Wang^*^

Department of Chemistry & Biochemistry, Rowan University, Glassboro, NJ

^⊥^Authors contributed equally.

*Corresponding Author. Email: wangz@rowan.edu


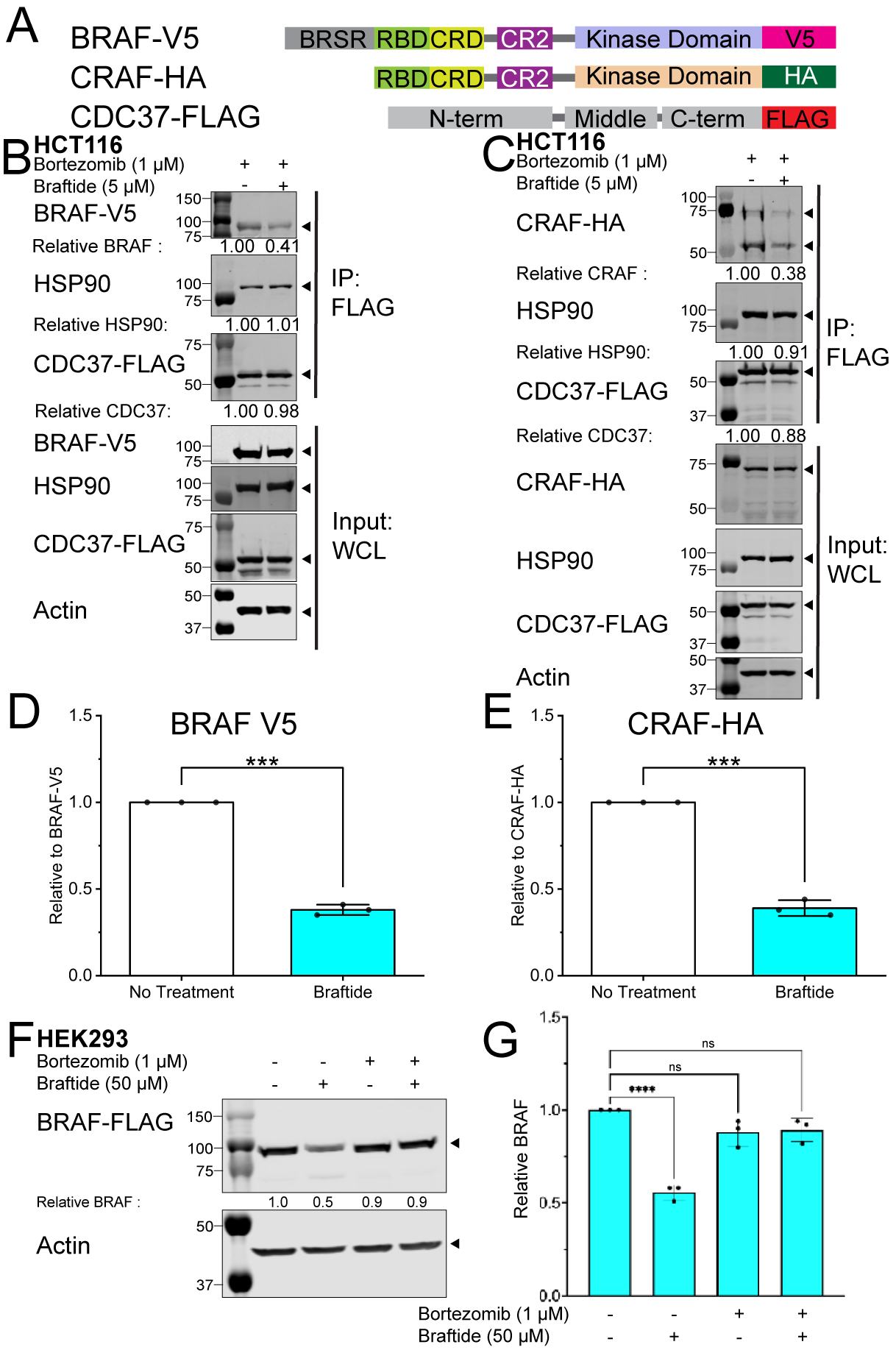


**Figure S1. Braftide disrupts the CDC37 chaperone complex in HCT116 cells.** A) Cartoon schematic of constructs used for probing the client RAF and CDC37 interaction B-C) Representative immunoblots of HCT116 cells exogenously expressing BRAF-V5 (B) or CRAF-HA (C) with CDC37-FLAG in the presence and absence of 5 µM Braftide. Immunoprecipitated CDC37-FLAG decreases its association with coimmunoprecipitated BRAF-V5 and CRAF-HA. D-E) Densitometry analysis of BRAF (D) and CRAF (E) association with CDC37 across three biological replicates. F) Representative immunoblot of overexpressed BRAF-FLAG in HEK293 cells treated with Braftide in the absence and presence of bortezomib. G) Densitometry analysis of BRAF levels in WCL across three biological replicates.


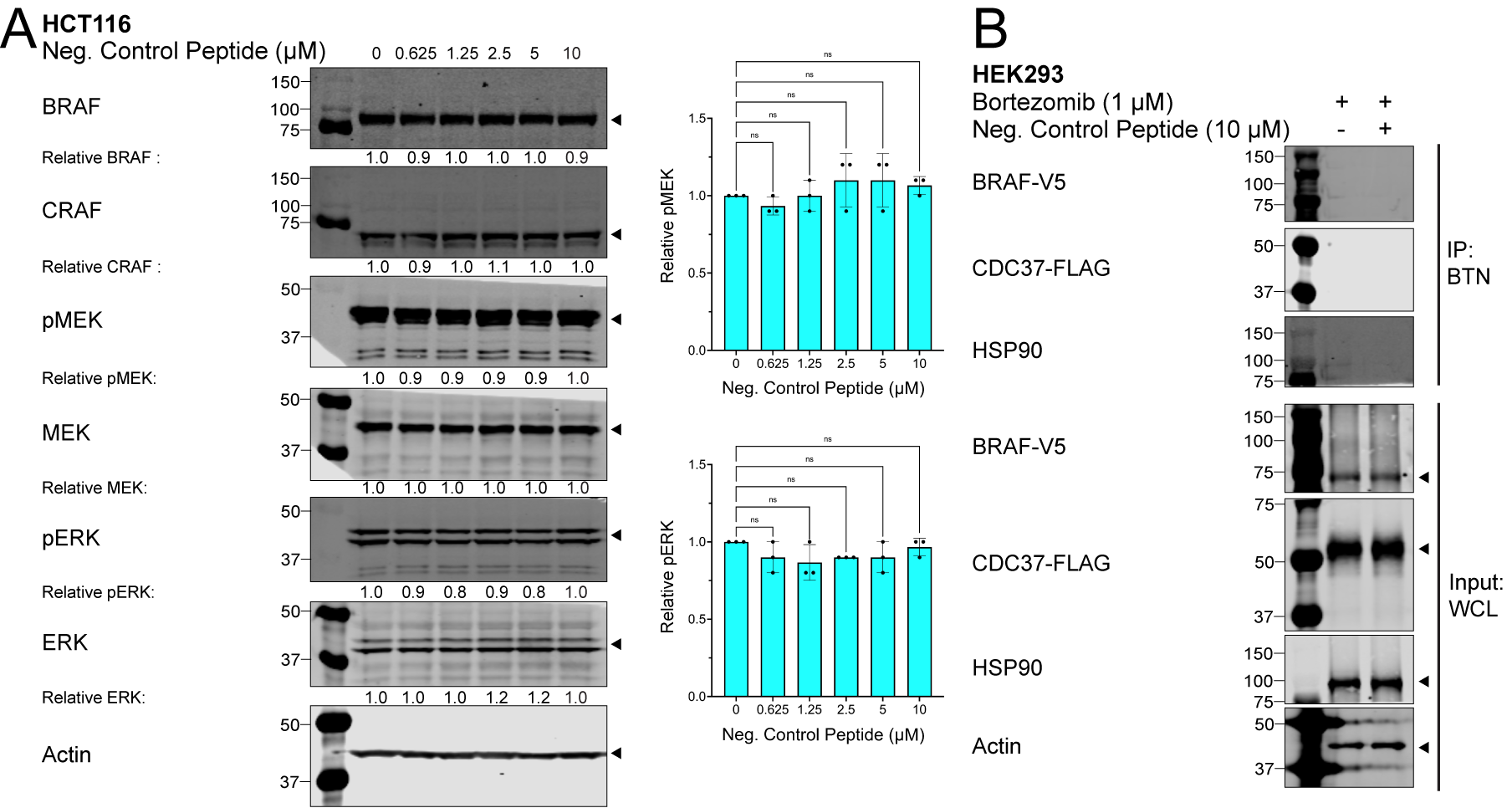


**Figure S2. MAPK proteins are unaffected by control peptide.** A) HCT116 cells were treated with negative control peptide at the indicated concentrations for 4 hrs. The cells were then harvested, lysed, and immunoblotted for MAPK proteins. Densitometry analysis of pMEK and pERK normalized to no treatment (0 μM) control peptide. B) Representative immunoblot of overexpressed BRAF-V5 and CDC37-FLAG in HEK293 cells in the absence and presence of crosslinked 10µM BTN-control peptide then immunoprecipitated and probed for MAPK proteins. At least three independent biological replicates were performed for each experiment.


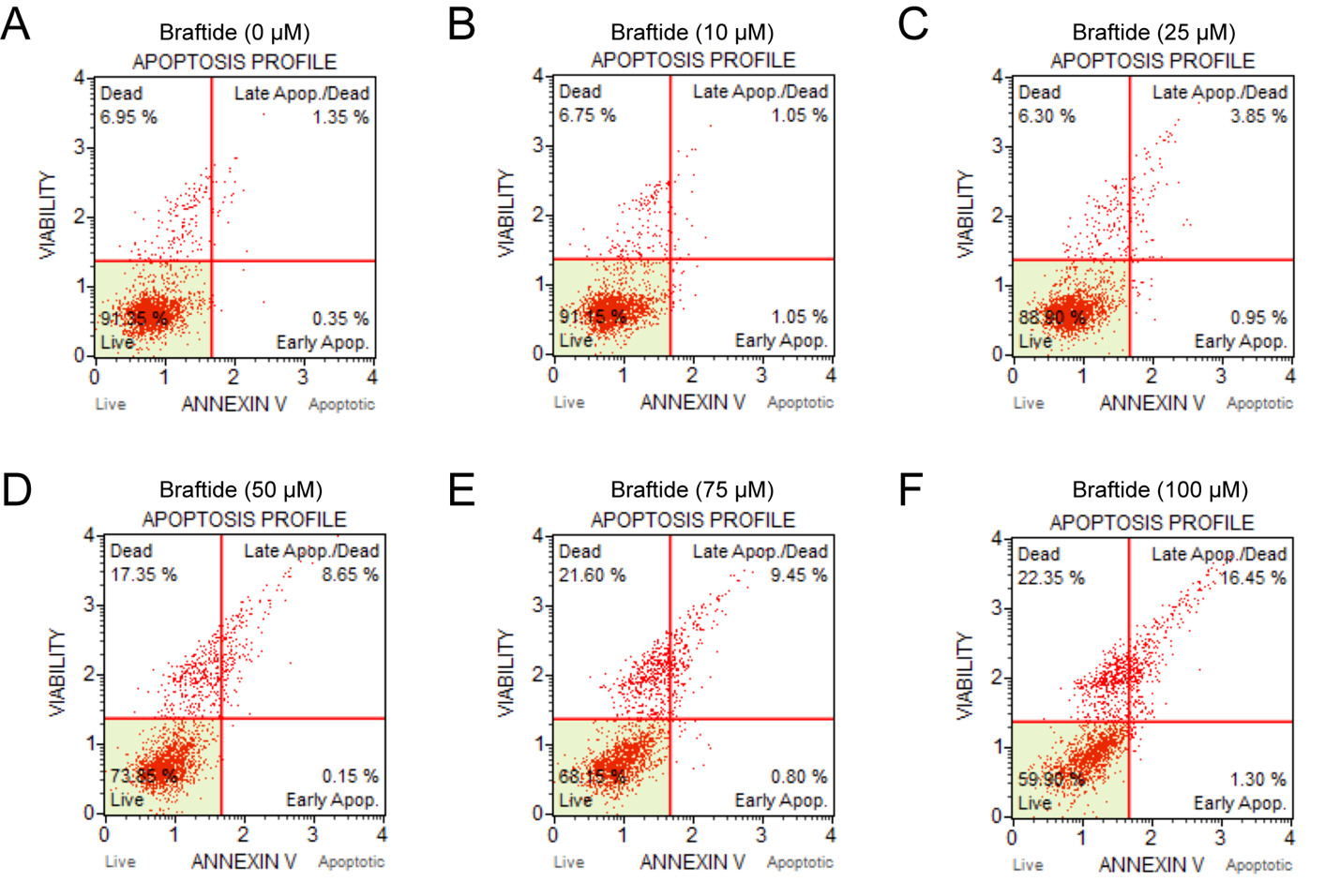


**Figure S3. Braftide treatment triggers apoptosis**. A-F) Representative apoptosis profile of HEK293 cells treated with Braftide at 0 (A), 10 (B), 25 (C), 50 (D), 75 (E), and 100 µM (F) for 4 hrs.


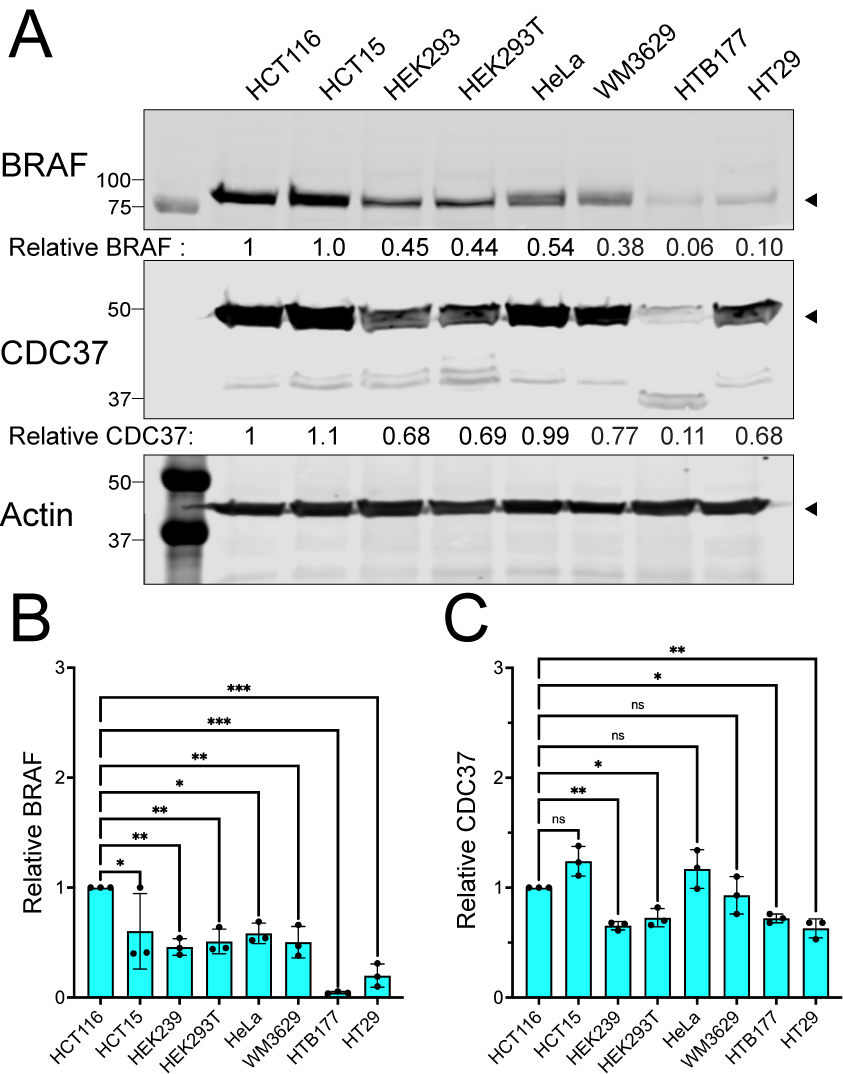


**Figure S4. HCT116 expresses high BRAF and CDC37 across several cell lines**. A) Representative immunoblot of several cell lines probed for endogenous BRAF and CDC37 across three biological replicates B-C) Densitometry analysis of BRAF (B) and CDC37 (D) across three biological replicates (n=3). Graph bars represent the mean ± SD with corresponding P-values (*P<0.05, **P<0.01, ***P<0.001). Statistical significance was determined via one-way ANOVA, followed by the post-hoc Tukey’s test.


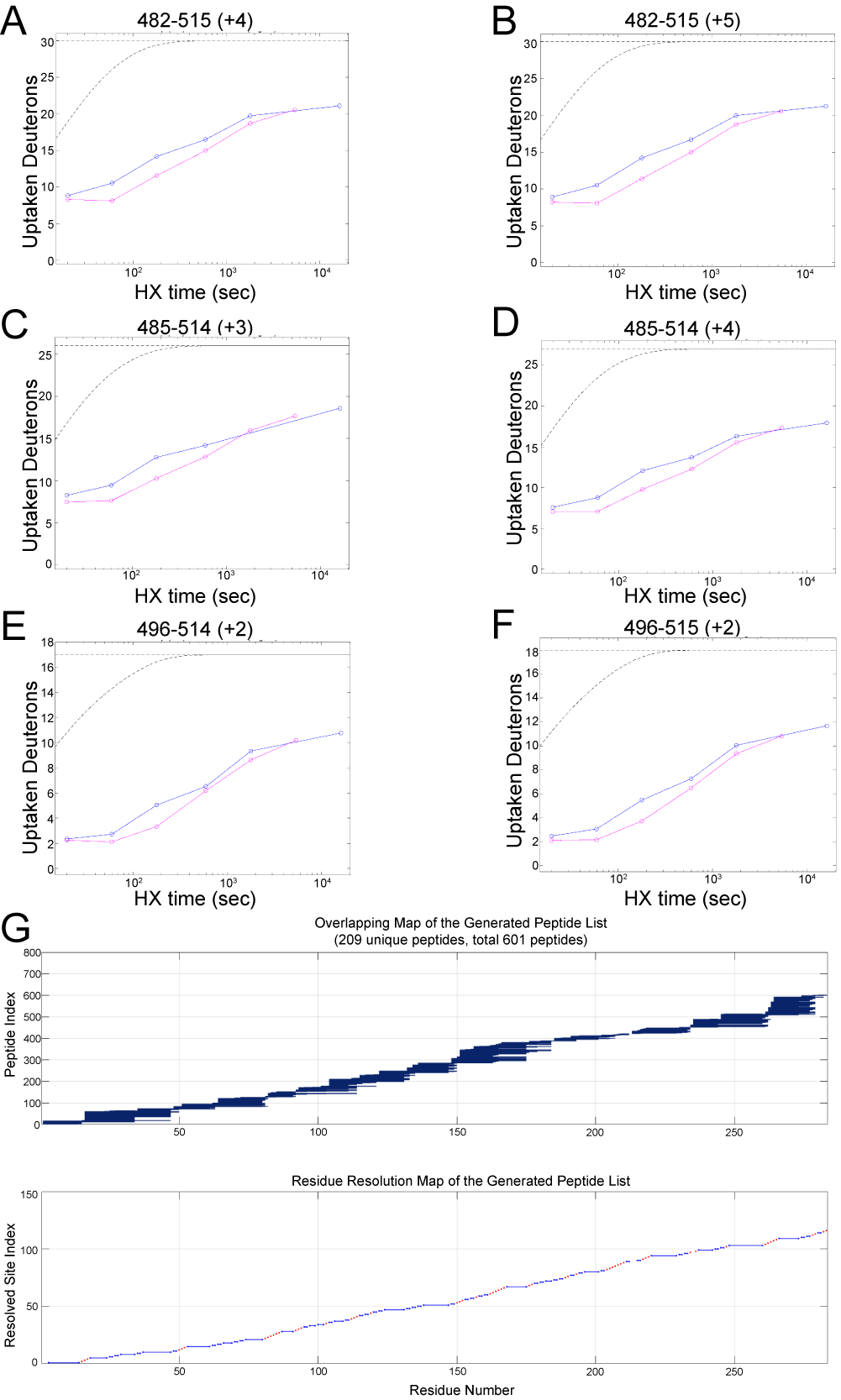


**Figure S5. Replicate HDX-MS data validate Braftide effect on deuterium uptake in the DIF region of BRAF^KD^.** A–F) Representative deuterium uptake plots for peptides within the DIF of BRAF^KD^ from an independent replicate HDX-MS experiment. Consistent with the first replicate (Figure 3), Braftide (pink) slows deuterium incorporation compared to apo BRAF^KD^ (blue), indicating reproducible protection in the DIF region upon Braftide binding. Gray dashed lines denote theoretical exchange profiles: the upper line represents a fully unstructured peptide, and the lower line represents complete hydrogen-bond protection. G) Peptide coverage map of BRAF^KD^ showing 98% sequence coverage for both replicates.


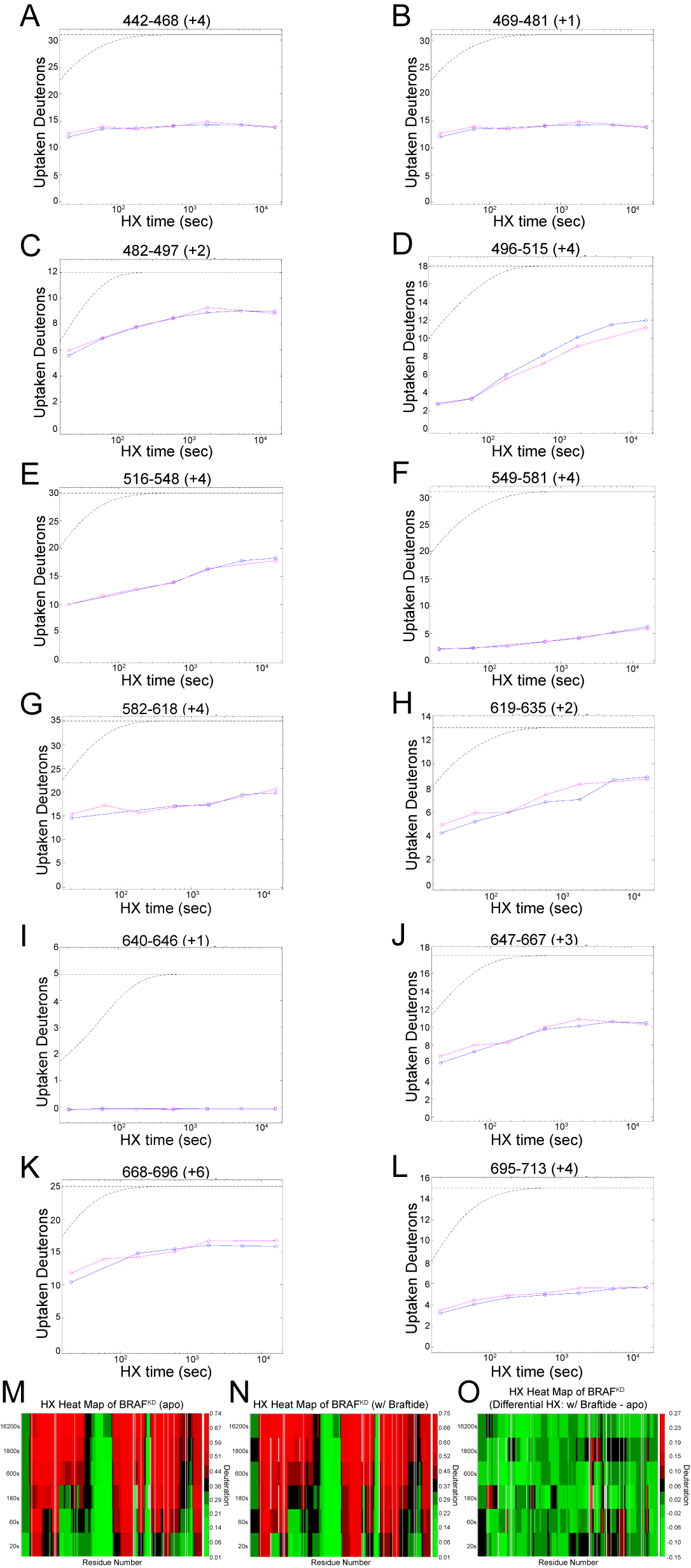


**Figure S6. Braftide slows deuterium uptake in the DIF region of BRAF.** A-L) Peptide plots spanning the entire BRAF^KD^ region that show essentially no change except in the dimer interface region, in the absence (blue) and presence (pink) of Braftide. Gray dashed lines represent theoretical exchange profiles: the upper line corresponds to a fully unstructured peptide, while the lower line represents a peptide with complete hydrogen bond protection. M-O) Heatmaps of: BRAF^KD^ in the absence of Braftide (M), BRAF^KD^ in the presence of Braftide (N), and the differential heatmap of apo BRAF^KD^ subtracted from BRAF^KD^ with Braftide (O).


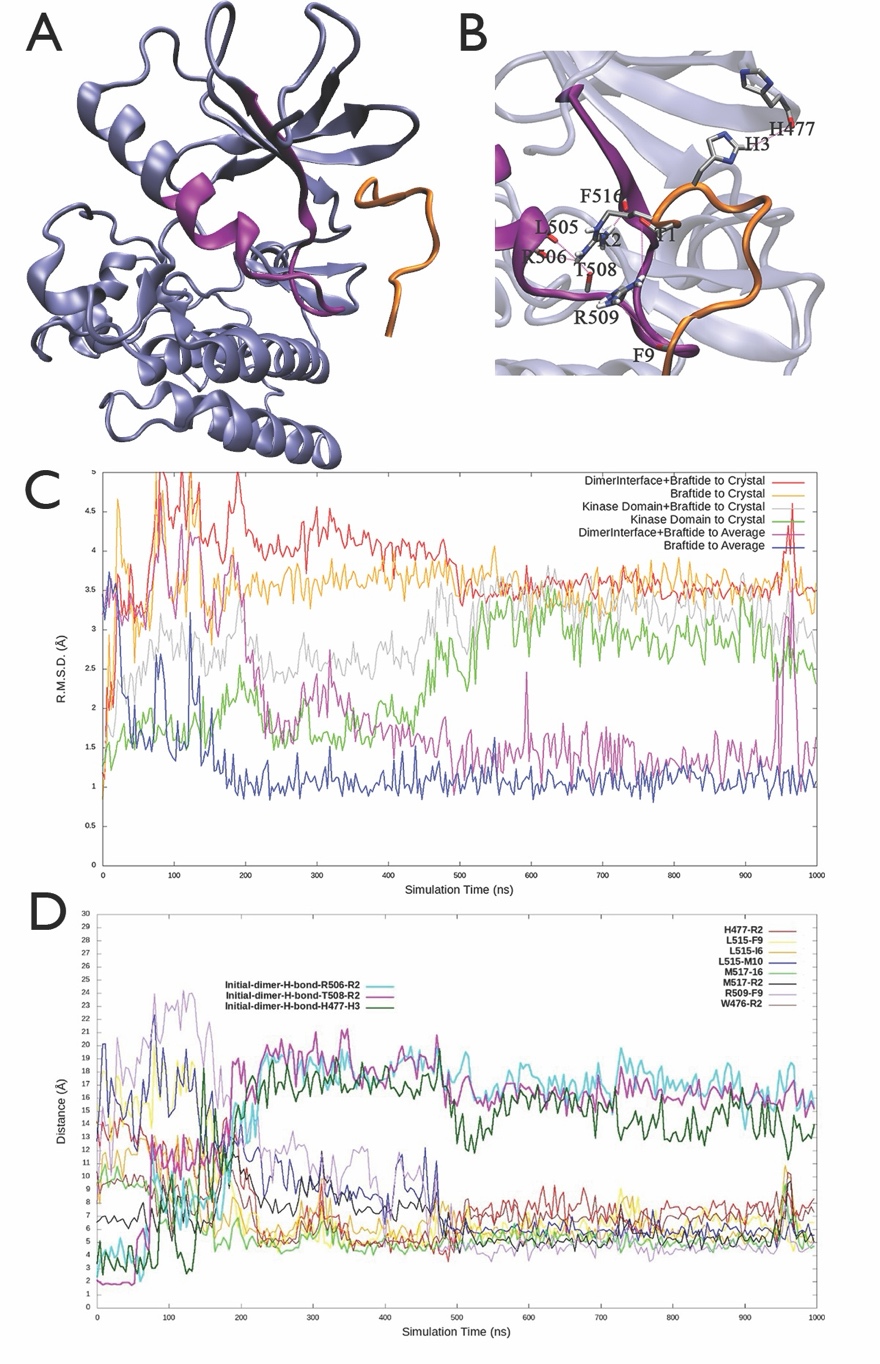


**Figure S7.** A) BRAF-Braftide MD simulation starting structure, extracted from the active BRAF kinase domain dimer (PDB: 4e26). B) Enlarged dimer interface of the starting structure in A) showing key hydrogen bonding interactions. C) RMSD plots of Ca atoms of different sets of residues with respect to either the starting structure (crystal) or the 200 ns to 1000ns average structure showing deviation from starting structure, relatively larger fluctuations from 0 to ~500 ns with Braftide exploring the conformational space, as well as convergence to a stable conformation from 500 ns to 1000ns. D) Selected residue-residue distance profile along the MD trajectory, showing BRAF-Braftide interaction is distinctly different from that of the dimer-like interaction, though involving similar group of residues (dimer interface).


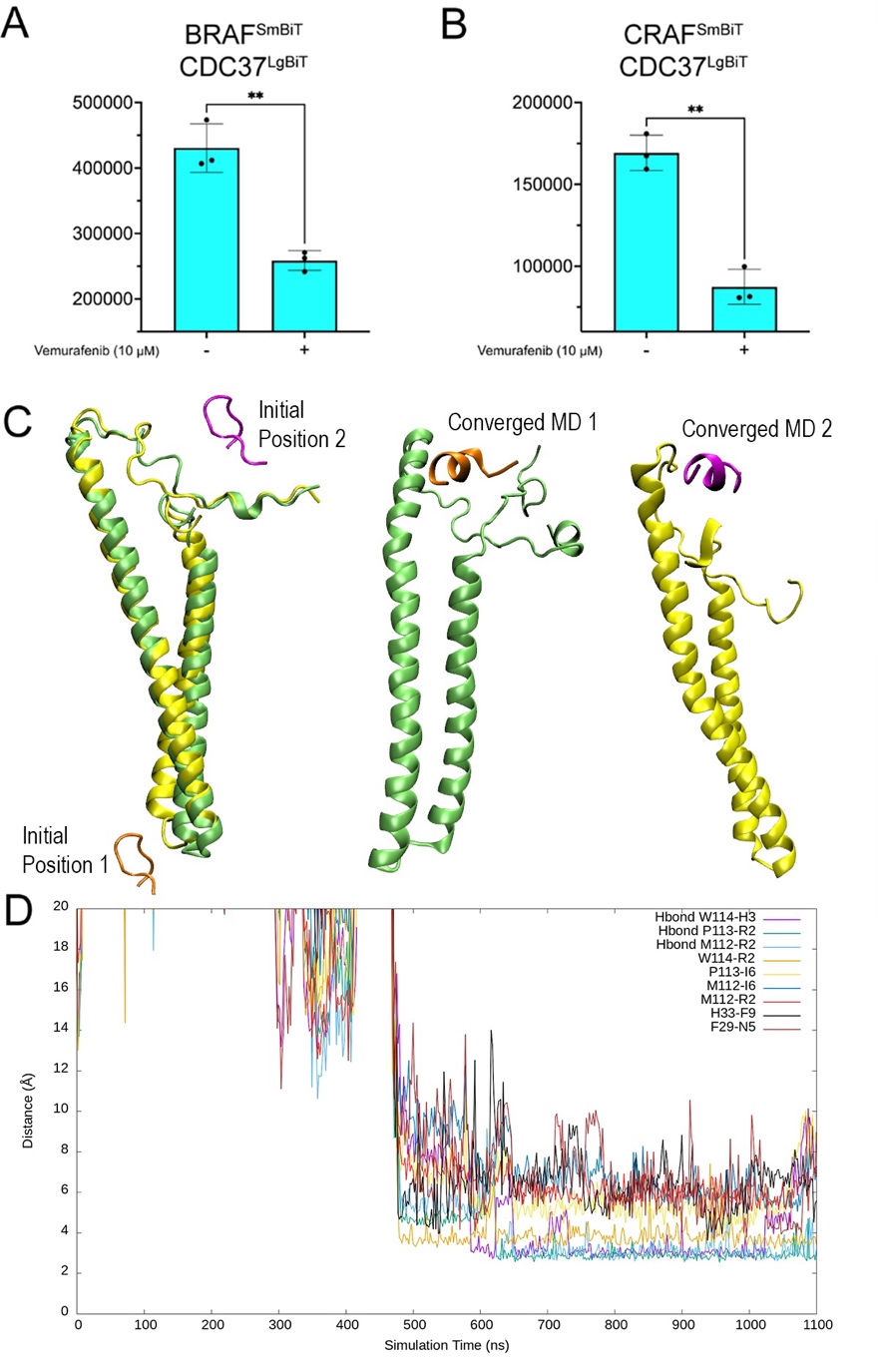


**Figure S8.** A-B) NanoBiT assay in the absence and presence of Vemurafenib (10 µM, 4hrs) in HEK293 cells expressing NanoBiT constructs of BRAF^SmBiT^-CDC37^LgBiT^ (A) and CRAF^SmBiT^-CDC37^LgBiT^ (B) normalized to no treatment control across three biological replicates. At least three independent biological replicates were performed for each experiment. C) MD starting structures and snapshots of converged binding modes for the two independent CDC37/Braftide simulations, green-orange for simulation 1 and yellow-magenta for simulation 2. D) Distances indicating hydrogen bonding and hydrophobic interaction between Braftide and CDC37 residue pairs along the trajectory of MD simulation 1. The plot shows a group of persistent hydrogen bonding and hydrophobic contacts after 500 ns indicating convergence to a stable binding mode. Simulation 2 shows similar convergence after 200 ns.


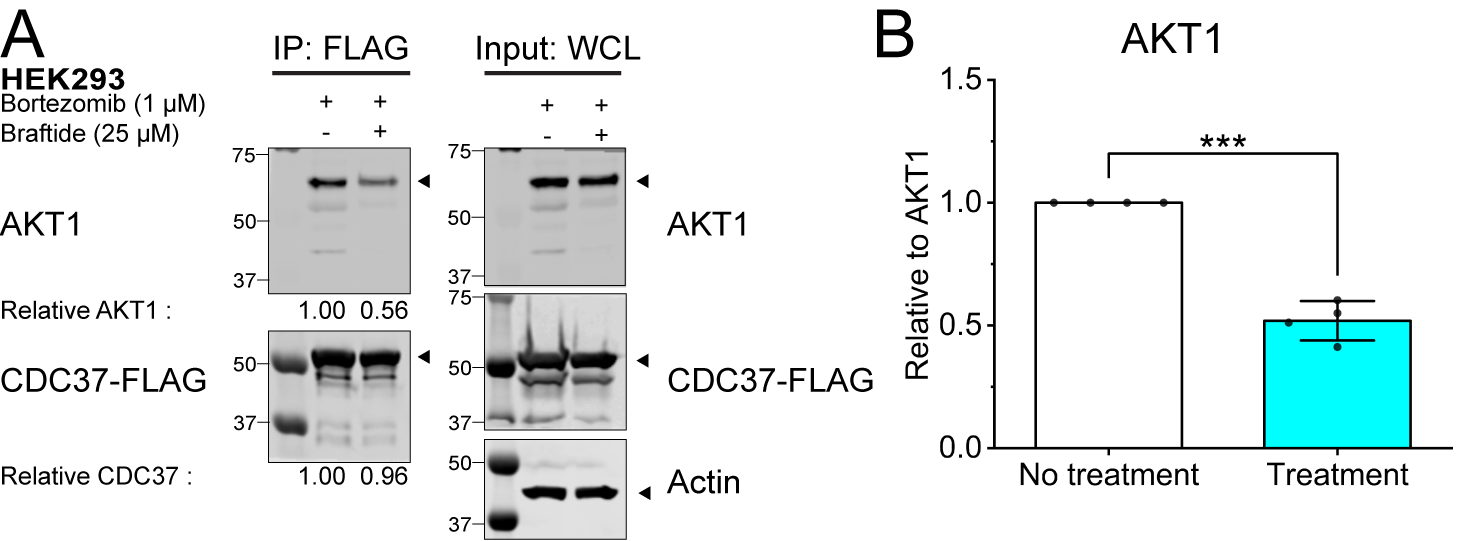


**Figure S9. Braftide treatment disrupts client AKT1 from CDC37-FLAG in HEK293 cells.** A) Representative immunoblot of immunoprecipitated CDC37 with co-immunoprecipitated AKT1 of three biological replicates in the absence and presence of Braftide (25 µM) treatment. Cells are bortezomib pretreated to prevent proteasomal degradation. B) Densitometry analysis of four biological replicates (n=4). Graph bars represent the mean ± SD with corresponding P-values (*P<0.05, **P<0.01, ***P<0.001). Statistical significance was determined via one-way ANOVA, followed by the post-hoc Tukey’s test.


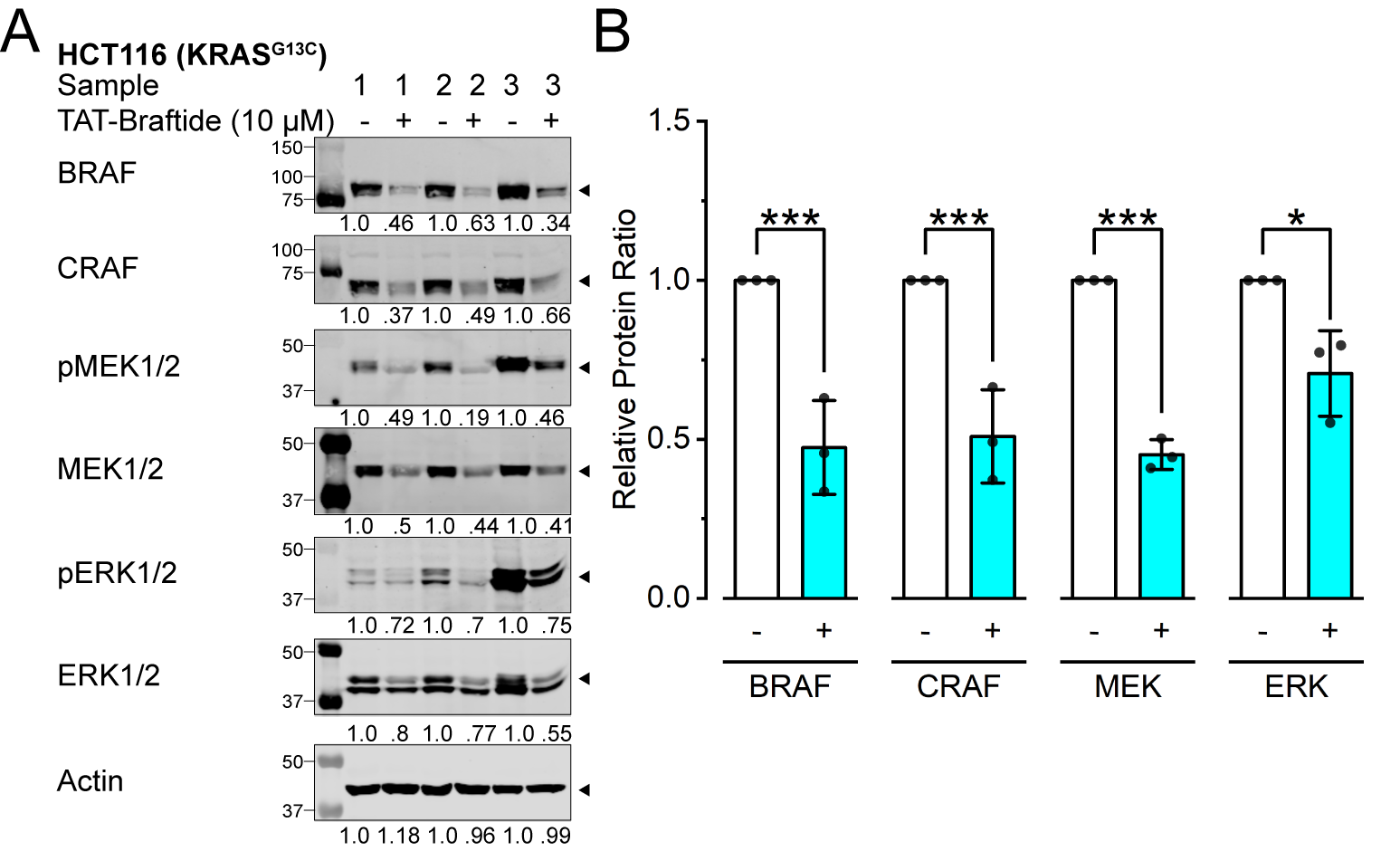


**Figure S10. Braftide decreases MAPK pathway protein levels in samples prepared for global proteomics.** A-B) Immunoblot of TAT-Braftide-mediated downregulation of RAF-MEK1/2-ERK1/2 pathway protein levels in HCT116 cells (constitutively active KRASG13C mutant cancer cell line) (n=3). KRAS G13C hyperactivates the RAF-MEK1/2-ERK1/2 pathway. These three biological replicates were subjected to LC-MS/MS for differential protein expression in the presence and absence of Braftide treatment (10 µM, 1 hr, 37°C). The relative ratio is relative to the non-treatment sample (no treatment/Braftide treatment). B/CRAF, phosphorylated MEK1/2 (pMEK1/2), total MEK1/2, phosphorylated ERK1/2 (pERK/2), total ERK1/2, and actin are visualized in the immunoblots. B) Densitometry analysis of the samples used for differential global proteomic identification of the MAPK pathway constituents. Statistical significance was determined via two-way ANOVA, followed by the post-hoc Tukey’s HSD (honest significant difference) test. Graph bars represent the mean ± SD with individual data points per biological replicate with corresponding P-values (*P<0.05, **P<0.01, ***P<0.001).


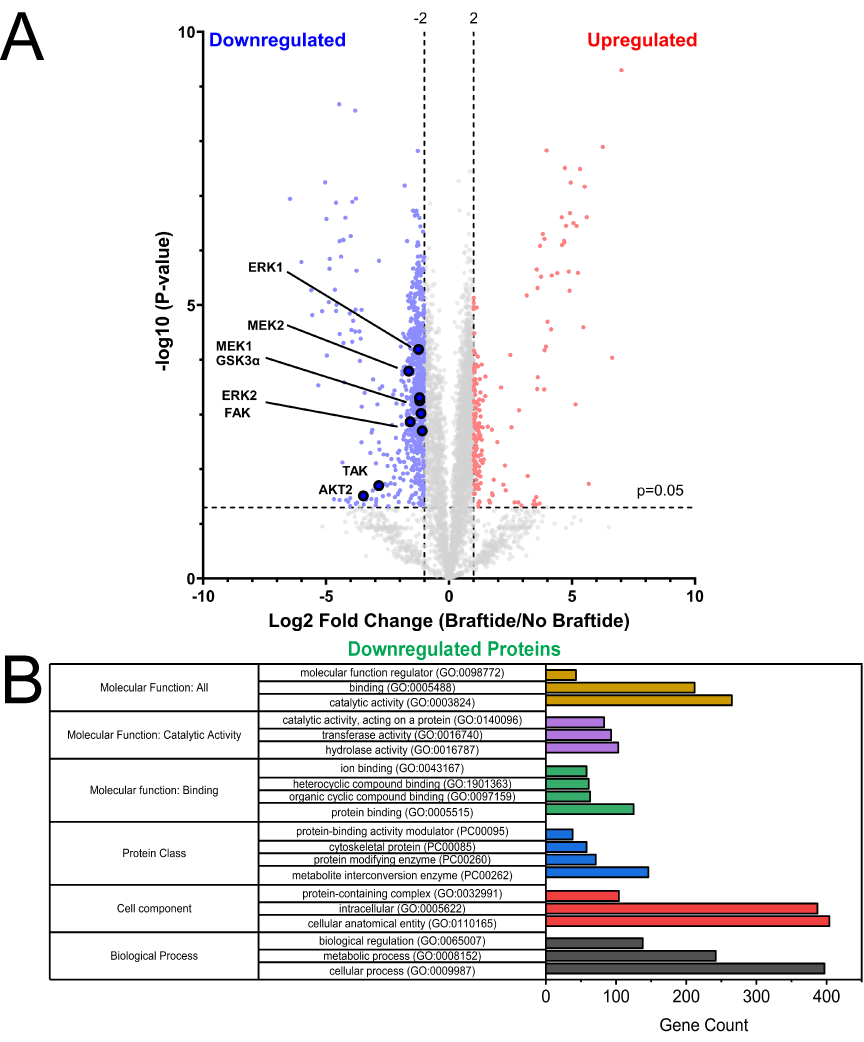


**Figure S11.** A) Volcano plot of 5405 proteins as a result of Braftide treatment identified in three biological replicates. The blue vertical lines represent the 2-FoldChange (FC) cutoff. Neon green dots represent the 627 proteins that are downregulated. Red dots represent the 118 upregulated proteins. B) Gene ontology (GO) analysis of downregulated proteins in which the top 3-4 most Braftide treatment enriched categories are graphed by gene count (frequency) using the Panther GO database.


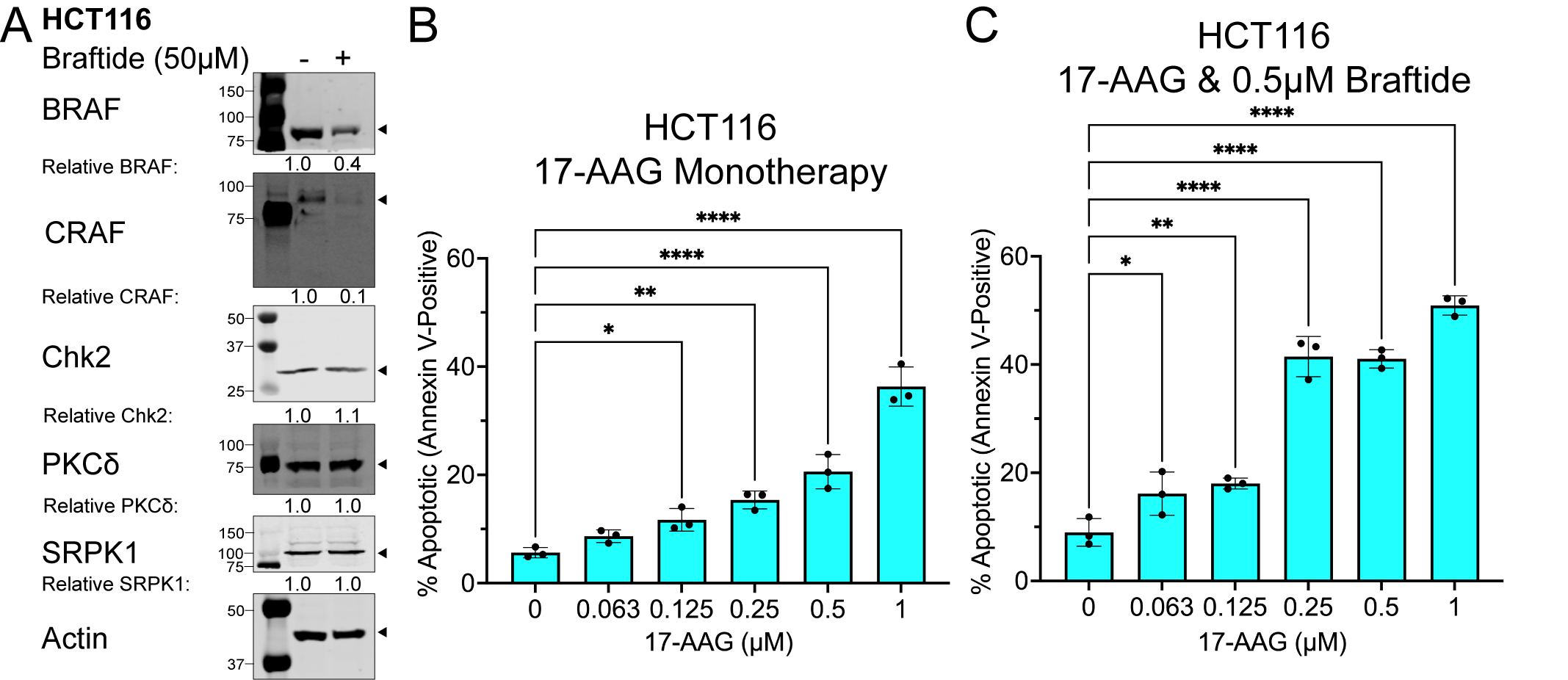


**Figure S12.** A) Braftide displays selectivity towards kinases reliant on HSP90-CDC37. Representative immunoblot of selected kinases that are unaffected by Braftide treatment. B-C) Braftide synergizes with 17-AAG in HCT116 cells to trigger cell death at lower treatment concentrations. Cells were stained with Annexin V, an apoptosis marker, and sorted via flow cytometry (n=3).

**Supplementary Information 1. Kinase Domain (KD) alignment of BRAF with the solubilizing mutations against full length (FL) BRAF.**

KD ------------------------------------------------------------ 0

FL MGHHHHHHMAALSGGGGGGAEPGQALFNGDMEPEAGAGAGAAASSAADPAIPEEVWNIKQ 60

KD ------------------------------------------------------------ 0

FL MIKLTQEHIEALLDKFGGEHNPPSIYLEAYEEYTSKLDALQQREQQLLESLGNGTDFSVS 120

KD ------------------------------------------------------------ 0

FL SSASMDTVTSSSSSSLSVLPSSLSVFQNPTDVARSNPKSPQKPIVRVFLPNKQRTVVPAR 180

KD ------------------------------------------------------------ 0

FL CGVTVRDSLKKALMMRGLIPECCAVYRIQDGEKKPIGWDTDISWLTGEELHVEVLENVPL 240

KD ------------------------------------------------------------ 0

FL TTHNFVRKTFFTLAFCDFCRKLLFQGFRCQTCGYKFHQRCSTEVPLMCVNYDQLDLLFVS 300

KD ------------------------------------------------------------ 0

FL KFFEHHPIPQEEASLAETALTSGSSPSAPASDSIGPQILTSPSPSKSIPIPQPFRPADED 360

KD ------------------------------------------------------------ 0

FL HRNQFGQRDRSSSAPNVHINTIEPVNIDDLIRDQGFRGDGGSTTGLSATPPASLPGSLTN 420

442

KD ----------------------MHHHHHH**G**TRDSSDDWEIPDGQITVGQRIGSGSFGTVY 38

FL VKALQKSPGPQRERKSSSSSEDRNRMKTL**G**RRDSSDDWEIPDGQITVGQRIGSGSFGTVY 480

:: : * *****************************

KD KGKWHGDVAVKMLNVTAPTPQQLQAFKNEVGVLRKTRHVNILLFMGYSTKPQLAIVTQWC 98

FL KGKWHGDVAVKMLNVTAPTPQQLQAFKNEVGVLRKTRHVNILLFMGYSTKPQLAIVTQWC 540

************************************************************

KD EGSSLYHHLHASETKFEMKKLIDIARQTARGMDYLHAKSIIHRDLKSNNIFLHEDNTVKI 158

FL EGSSLYHHLHIIETKFEMIKLIDIARQTAQGMDYLHAKSIIHRDLKSNNIFLHEDLTVKI 600

********** ****** **********:************************* ****

KD GDFGLATVKSRWSGSHQFEQLSGSILWMAPEVIRMQDSNPYSFQSDVYAFGIVLYELMTG 218

FL GDFGLATVKSRWSGSHQFEQLSGSILWMAPEVIRMQDKNPYSFQSDVYAFGIVLYELMTG 660

*************************************.**********************

KD QLPYSNINNRDQIIEMVGRGSLSPDLSKVRSNCPKRMKRLMAECLKKKRDERPSFPRILA 278

FL QLPYSNINNRDQIIFMVGRGYLSPDLSKVRSNCPKAMKRLMAECLKKKRDERPLFPQILA 720

************** ***** ************** ***************** **:***

KD EIEELARELSG------------------------------------------------- 289

FL SIELLARSLPKIHRSASEPSLNRAGFQTEDFSLYACASPKTPIQAGGYGAFPVH------ 774

.** ***.*

**Supplementary Information 2. Mass spectrometry data from Braftide treated HCT116 cells with differential protein expression (xls file).**
